# Pyruvate Kinase M2‐Mediated H3K9 Lactylation and XBP1 Signaling Positive Feedback Loop Facilitates CD4^+^ T Cell Spinal Recruitment and Central Sensitization in Rheumatoid Arthritis

**DOI:** 10.1002/advs.78397

**Published:** 2026-11-09

**Authors:** Yuepeng Jiang, Xiaoxuan Zhao, Xiao Ma, Mengjia Zheng, Junjun Wen, Yujie Li, Yue Wu, Yun Zhang, Jie Yu, Charles R. Ashby Jr, Chengping Wen

**Affiliations:** ^1^ College of Basic Medical Sciences Zhejiang Chinese Medical University Hangzhou Zhejiang China; ^2^ Department of Traditional Chinese Medicine (TCM) Gynecology Hangzhou Hospital of Traditional Chinese Medicine Affiliated to Zhejiang Chinese Medical University Hangzhou Zhejiang China; ^3^ Department of Pharmaceutical Sciences College of Pharmacy and Health Sciences St. John's University Queens New York USA

**Keywords:** CD4^+^ T cells, central sensitization, histone lactylation, microglia, PKM2, rheumatoid arthritis, XBP1

## Abstract

Central sensitization is a critical pathophysiological mechanism underlying chronic pain in rheumatoid arthritis (RA). CD4^+^ T cells not only promote chronic inflammation in RA but may also migrate into the central nervous system to stimulate central sensitization. However, the regulatory mechanism by which CD4^+^ T cells contribute to central sensitization in RA remains unclear. In this study, we report that CD4^+^ T cells were significantly accumulated in the spinal dorsal horn (SDH) of collagen‐induced arthritis (CIA) mice and promoted their central sensitization‐like behaviors. Mechanistically, PKM2‐dependent lactate facilitated H3K9 lactylation (H3K9la) in CD4^+^ T cells. H3K9la was highly enriched at the promoters of XBP1, which facilitated the transcription of *KIF4* and *MYO1B* and the accumulation of CD4^+^ T cells in SDH. Furthermore, XBP1 may facilitate the transcription of PKM2, establishing a positive feedback loop between PKM2‐induced H3K9la and XBP1 signaling in CIA mice. Crucially, the clinical relevance of this pathway was validated in patients with RA, where the expression of PKM2/H3K9la/XBP1 was significantly increased and positively correlated with central sensitization scores. Overall, our study indicates a positive feedback loop of PKM2/H3K9la/XBP1 in facilitating CD4^+^ T cell spinal recruitment, which may provide a promising therapeutic strategy for mitigating central sensitization in RA.

## Graphical abstract

In collagen‐induced arthritis (CIA) mice, increased CD4^+^ T cell accumulation in the spinal dorsal horn induces pain hypersensitivity. Mechanistically, PKM2‐mediated H3K9 lactylation and XBP1 signaling positive feedback loop facilitates CD4^+^ T cell spinal migration and induces central sensitization‐like behaviors in CIA mice. This clinically relevant pathway offers a promising therapeutic target for chronic pain in rheumatoid arthritis.

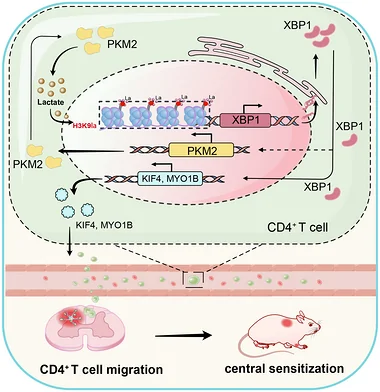

**Abbreviations**
ChIP: chromatin immunoprecipitation
CIA: collagen‐induced arthritis
CNS: central nervous system
CSI: central sensitization inventory
CUT&Tag: cleavage under targets and tagmentation
GO: gene ontology
H3K9la: H3K9 lactylation
KEGG: kyoto encyclopedia of genes and genomes
KIF4: kinesin family member 4
MLC: myosin light chain
MYO1B: myosin IB
PKM2: pyruvate kinase M2
RA: rheumatoid arthritis
RhoA: Ras homolog family member A
SDH: spinal dorsal horn
XBP1: X‐box binding protein 1

## 1 Introduction

Rheumatoid arthritis (RA) is the most common type of inflammatory arthritis, with a global prevalence of 0.5% to 1% [1]. Chronic pain is the most universal and refractory symptom in RA [2]. As many as 11.9% of individuals with RA report residual chronic pain as a major unmet medical need, despite optimal control of joint inflammation [3]. This raises concerns about the multifactorial nature of pain in RA extending beyond inflammation. Central sensitization is a pathophysiological mechanism characterized by heightened neuronal reactivity within central pain pathways following injury [4]. Approximately 40% of individuals with RA exhibit central sensitization syndrome [5]. However, the mechanism by which central processing mediates chronic pain in RA remains to be determined.

Microglia are significant neuromodulators that induce chronic pain [6]. Once activated, microglia produce cytokines, chemokines, and neuropeptides, which facilitate synaptic transmission, neuronal excitability, and central sensitization maintenance [7, 8]. Microglial activation is one of the main inducers of central sensitization in a variety of chronic pain states, including experimental arthritis models [9], but its potential mechanisms and specific involvement in RA‐associated pain remain fully uncharacterized. It has been reported that the stimulation of glial cells by circulating monocytes promotes chronic pain in autoimmune diseases [10]. Resident immunocompetent glial cells in the central nervous system (CNS) not only signal to immune cells but also respond to inflammatory mediators [11]. Of all circulating leukocytes, CD4^+^ T cells exhibit a profound migratory capacity, continuously trafficking between the lymph and blood, and infiltrating sites of inflammation [12]. Under pathological conditions, auto‐reactive CD4^+^ T cells localize within CNS inflammatory lesions [13] and emerge as the most potent leukocyte subpopulation for activating glial cells and inducing central sensitization [14]. Genetic deletion of the CD4 gene significantly blocks CD4^+^ T cell‐induced glial activation, consequently decreasing allodynia and thermal hyperalgesia [15]. Notably, our prior studies have reported central sensitization‐like behaviors in a collagen‐induced arthritis (CIA) murine model, which were accompanied by increased polarization of CD4^+^ T cells with enhanced migratory properties driven by dynamic cytoskeletal remodeling [16, 17]. However, the upstream mechanisms that regulate this T‐cell migration and its downstream effects on microglial activation during the development of central sensitization in RA remain to be determined.

Patients with RA exhibit metabolic disturbances [18]. The inflammatory microenvironment within arthritic joints is typically hypoxic, which contributes to metabolic imbalance and lactate accumulation [19]. Moreover, metabolic reprogramming is closely implicated in the inflammatory response of RA, wherein accelerated glycolysis fuels the pro‐inflammatory phenotype of macrophages [20]. Likewise, T cells in RA exhibit a distinct metabolic profile compared with those from healthy individuals [21]. Enhanced glycolysis drives T cell dysregulation in RA [22]. A recent study reported that CD4^+^ T cell migration depends heavily on glucokinase‐mediated glycolysis [23]. In patients with RA, activated T cells shift their main energy source from oxidative phosphorylation to glycolysis [24]. In addition to providing a rapid source of ATP, glycolysis produces metabolites that modulate immune cell function by post‐translational modification of lactylation [25]. Lactate has recently been identified as a signaling molecule that induces an epigenetic alteration, known as histone lysine lactylation (Kla) [26, 27]. This histone modification at the lysine residues of histone tails affects chromatin packaging and accessibility, thus directly promoting gene transcription [28]. Histone lactylation serves as a pivotal bridge linking metabolic reprogramming to the pathogenesis of autoimmune diseases, including RA [29]. Interestingly, this modification functions as a vital epigenetic regulator during pathogenesis by facilitating cell migration [30, 31]. However, the interplay between metabolic profile, histone lactylation, and CD4^+^ T cell migration in RA warrants further investigation.

Therefore, we established that the accumulation of circulating CD4+ T cells into the CNS is critical for driving microglial activation and central sensitization‐like behaviors in a murine CIA model. Pyruvate kinase M2 (PKM2)‐dependent aerobic glycolysis, which catalyzes the biotransformation of glucose to lactate, is essential for the spinal recruitment of these cells. Furthermore, our study indicated that H3K9la levels are significantly increased, which contributed to the transcription of the X‐box binding protein 1 (XBP1). XBP1 directly binds to the kinesin family member 4 (KIF4) and myosin IB (MYO1B) promoters and increases their expression, thus facilitating the dynamic remodeling involved in cell migration and invasion. Intriguingly, XBP1 reciprocally up‐regulated PKM2 expression. Overall, our data revealed a positive feedback loop, comprising PKM2‐dependent glycolysis, H3K9la, and XBP1, which drives CD4^+^ T cell trafficking into the CNS. Furthermore, clinical data confirmed the relevance of this PKM2/H3K9la/XBP1 axis to central sensitization in patients with RA. Overall, these findings may facilitate the development of novel treatments for pain relief in RA.

## 2 Materials and Methods

All materials used in this study are listed in Table S1.

### 2.1 Model Preparation and Animal Grouping

#### 2.1.1 Experimental Animals

Specific‐pathogen‐free (SPF) male DBA/1 mice were provided by Shanghai SLAC Animal Co., LTD. PKM^fl/fl^ mice (Cat. NO: NM‐CKO‐200206, Shanghai Model Organisms Center, Inc.) and CD4‐Cre mice (T004818, Gempharmatech Company) were used to generate CD4‐Cre^+/−^PKM^fl/fl^ conditional knockout and littermate control CD4‐Cre^−/−^PKM^fl/fl^ mice (WT). Laboratory animals (aged 6–8 weeks, 20 ± 2 g) were kept in the Laboratory Animal Center (LARC) of Zhejiang Chinese Medical University under a controlled environment (21±1°C; 55±5% humidity; 12‐h light/dark cycle). All animal procedures were performed in accordance with the guidelines approved by the Institutional Animal Care and Use Committee (IACUC) of Zhejiang Chinese Medical University (Approval No.: IACUC‐202311‐26).

#### 2.1.2 Establishment of a CIA Mouse Model

Mice were acclimatized until 8 weeks of age, at which point the collagen‐induced arthritis model was initiated. To prepare the emulsions, a bovine type II collagen solution (dissolved in glacial acetic acid) was homogenized with an equal volume of either Freund's complete adjuvant (4 mg/mL) for the initial sensitization or Freund's incomplete adjuvant for the booster. On day 0, each mouse received a 100 µL injection of the primary emulsion at the tail base. On day 21, the emulsion was injected for booster immunization (100 µL per mouse). Based on our previously published study [16], day 42 post‐primary immunization was designated as the chronic pain stage, which is characterized by established central sensitization‐like behaviors and a significant decrease in the pain threshold. Paw arthritis severity in CIA mice was assessed using a 16‐point arthritic index (4 points/limb) [32].

### 2.2 Experimental Design and Treatment Strategies

(A) DBA/1 mice were randomly divided into two groups (*n* = 8 per group): (1) normal control and (2) CIA model.

(B) DBA/1 mice were randomly divided into two groups (*n* = 8 per group): (1) CIA+anti‐CD4 antibody and (2) CIA+control antibody. Every 7 days, CD4^+^ T cells were depleted by intravenously injecting 500 µg of an anti‐CD4 antibody.

(C) DBA/1 mice were randomly divided into two groups (*n* = 8 per group) to determine the effect of lactate on CD4^+^ T cell migration: (1) CIA+sodium oxamate (oxamate) and (2) CIA+vehicle. Oxamate (750 mg/kg/day) [33, 34], a lactate dehydrogenase inhibitor that suppresses glycolytic lactate production, was administered by intraperitoneal injection.

(D) DBA/1 mice were randomly divided into two groups (*n* = 8 per group) for in vivo histone lactylation blockade: (1) CIA+vehicle and (2) CIA+C646. C646 (10 mg/kg) was given by intraperitoneal injection.

(E) CD4‐Cre mice were randomly divided into two groups (*n* = 8 per group) to determine the effect of LDHA knockdown on CD4^+^ T cell recruitment via AAV‐ARK313‐DIO vectors: (1) CD4^Cre^+scramble shRNA+CIA and (2) CD4^Cre^+LDHA shRNA+CIA. Mice were injected intravenously with the respective AAV vectors at a dose of 2.5 × 10^11^ vg/mouse.

(F) CD4‐Cre mice were randomly divided into three groups (*n* = 8 per group) for in vivo rescue experiments via AAV‐ARK313‐DIO vectors: (1) CD4^Cre^+scramble shRNA+CIA+NC; (2) CD4^Cre^+PKM2 shRNA+CIA+NC; and (3) CD4^Cre^+PKM2 shRNA+CIA+XBP1 OE. Mice were injected intravenously with the respective AAV vectors at a dose of 2.5 × 10^11^ vg/mouse.

(G) Mice were randomly divided into four groups (n = 8 per group) to determine the effect of PKM2 on the expression of H3K9la and XBP1 in CD4^+^ T cells: (1) control; (2) CIA; (3) WT+CIA, and (4) CD4^Cre^Pkm2^fl/fl^+CIA.

### 2.3 Mechanical Pain Threshold

Von Frey hairs (0.04 g, 0.07 g, 0.16 g, 0.4 g, 0.6 g, 1.0 g, 1.4 g, and 2.0 g) were used in the mice. Normal mice were tested beginning with 0.4 g. A positive nociceptive response (such as paw retraction, flicking, or violent shaking) prompted the use of the next lower force of the von Frey hair. In the absence of such behavior, the next level of the von Frey hair was applied. The pain threshold was determined using the simplified up‑down method [35].

### 2.4 Heat Pain Threshold

Mice were placed on a hot metal plate maintained at 50.0 ± 0.5°C. The paw withdrawal latency was defined as the time from the start of the test until the animal exhibited a pain response, such as jumping, paw withdrawal, or paw licking. To avoid tissue injury caused by thermal stimulation, the maximum cutoff time of 30 s was enforced.

### 2.5 Single‐Cell RNA Sequencing

#### 2.5.1 Sample Preparation

Fresh spinal cord tissues were immediately dissociated into a single‐cell suspension. The tissues were washed with Dulbecco's PBS and digested at 37°C for 15–25 min with collagenase II (2 mg/mL), collagenase IV (2 mg/mL), and dispase (0.2 mg/mL). After digestion, the solution was transferred through a 40 µm cell strainer into a centrifuge tube. The samples were centrifuged at 400 g for 5 min at 4°C to pellet the cells. Red blood cells were then removed. After acridine orange/propidium iodide staining, cell concentration and viability were determined using a Cellometer Auto 2000.

#### 2.5.2 Library Construction and Sequencing

Single‐cell transcriptomic library synthesis was performed according to the Chromium Next GEM Single Cell 3ʹ Reagent Kits v3.1 (10× Genomics) manual. Briefly, each cell sample was adjusted to a concentration of 1,000 cells/µL. The cell suspension was applied to the Chromium Controller to generate single‐cell gel beads‐in‐emulsions (GEMs), whereby individual cells were encapsulated with gel beads into droplets. Within the GEMs, cells were lysed, and their mRNA was barcoded during reverse transcription. Reverse transcription of GEMs was conducted on the Veriti 96‐well thermal cycler (Thermo Fisher Scientific, Waltham, MA, USA). Finally, a sample index PCR was conducted, and the final purification was performed using the SPRIselect beads. The libraries were sequenced on the NovaSeq 6000 platform (Illumina).

#### 2.5.3 Data Processing

The gene expression matrix derived from the selected cells was log‐normalized using the NormalizeData function in Seurat. We used the FindVariableFeatures function to obtain the top 2,000 highly variable genes. Following principal component analysis dimensionality reduction, the main cell clusters were identified using a method based on the Louvain algorithm. We then applied the FindAllMarkers function to detect marker genes for each cluster. These gene markers guided the annotation of cell types. The cell‐cell interaction network was inferred using CellChat v1.5.0.

### 2.6 Flow Cytometry

Collected spinal dorsal horn (SDH) tissues were dissected, and single‐cell homogenates were prepared using the tissue dissociation kit (Miltenyi Biotec) in a shaking incubator at 37°C for 30 min. The suspension was passed through a 70 µm cell strainer and resuspended in 36% Percoll. After centrifugation, the precipitated cells were collected and washed twice with 5 mL of PBS. Cells were incubated with 2 µL of Fc block for 10 min in the dark. The appropriate amount of cell surface fluorescent antibodies (APC‐Cy7 anti‐CD45, PB anti‐CD3, FITC anti‐CD4) was added and incubated at 4°C for 40 min away from light. Next, the cells were washed with 1 mL of Stain Buffer and centrifuged at 400 × g for 5 min. Cells were resuspended in 300 µL of staining buffer and stained with 2 µL of 7‐Aminoactinomycin D. Flow cytometry was performed using a Beckman Coulter instrument (CytoFlex S, Beckman, CA, USA). Data were analyzed with FlowJo software.

### 2.7 Untargeted Metabolomics Analysis

Approximately 2 × 10^6^ cells per sample were snap‐frozen in liquid nitrogen, followed by the addition of 500 µL of ice‐cold extraction solvent. The mixture was vortexed for 30 s, incubated at −20°C for 1 h, and centrifuged at 16,000 × g for 15 min at 4°C. The metabolite‐containing supernatant was vacuum‐concentrated to dryness, stored at −80°C, and reconstituted in 100 µL of 2% acetonitrile in water before analysis. Chromatographic separation was performed on a Thermo UHPLC system coupled to a mass spectrometer (Thermo Fisher Scientific).

### 2.8 Metabolite Identification

The raw data files were converted to the mass spectrometry XML format, using the msConvert tool from ProteoWizard software. Following this, data were processed using the xCMS package. Metabolite annotation was performed by matching the accurate m/z values and MS/MS fragmentation patterns to public databases, with a mass accuracy threshold of 5 ppm.

The resulting feature matrix was imported into MetaboAnalyst 5.0 for statistical analysis. The data were normalized to the signal intensity of the quantity‐controlled samples and scaled to a Pareto chart. An Orthogonal Partial Least Squares Discriminant Analysis (OPLS‐DA) model was constructed to discern metabolite profiles between the control and CIA groups. Differential metabolites were selected at a Variable Importance in Projection (VIP) score > 1.0 and a *p*‐value from Student's *t*‐test < 0.05. These metabolites were then enriched via MetaboAnalyst to identify key metabolic pathways.

### 2.9 Detection of Protein Expression by Western Blot

Sodium dodecyl sulfate‐polyacrylamide gel electrophoresis was used to separate proteins from CD4^+^ T lymphocytes, followed by electrotransfer onto nitrocellulose membranes at 280 mA for 80 min. The membranes were incubated overnight with the primary antibodies (dilution of 1:1000) against PKM2, XBP1s, pan‐Kla, H3K9la, H3K14la, H3K18la, H3K23la, H3, phosphorylated myosin light chain (pMLC) Ser‐19, Ras homology gene family member A (RhoA), ionized calcium binding adapter protein1 (IBA1), and β‐actin. After washing, samples were incubated with secondary antibodies: goat anti‐rabbit IgG heavy and light chain (H&L) (1:5000) and goat anti‐mouse IgG (H&L) (1:5000) for 1 h at room temperature. The protein bands were then scanned using the Odyssey Infrared Imaging System and quantified using ImageJ software.

### 2.10 Immunofluorescence

The L4‐L6 spinal cord segments were fixed in 4% paraformaldehyde overnight and treated with 20% and 30% sucrose solutions sequentially. Frozen sections with a thickness of 12 µm were prepared. The Olympus VS120‐S6‐W digital pathology slide scanner (fluorescence) was used for imaging. The expression levels of interferon regulatory factor 8 (IRF8), IL‐1β, IBA1, PKM2, XBP1s, pan‐Kla, H3K9la, and CD4 in the SDH were determined (all antibodies diluted at 1:200).

### 2.11 CD4^+^ T Cell Isolation and Stimulation

Harvested spleen tissues were mechanically dissociated and suspended in RPMI 1640 medium. After erythrocyte depletion using a lysis buffer, single‐cell suspensions were obtained by filtering through a 70 µm filter. The cells were subsequently incubated with a magnetic activated cell sorting (MACS) buffer and a biotin antibody cocktail for 5 min, followed by incubation with anti‐biotin microbeads for 10 min at 4°C. Then, CD4^+^ T lymphocytes were isolated. Twenty‐four‐well plates were coated with anti‐CD3 antibody (10 µg/mL) in PBS. Splenic CD4^+^ T lymphocytes were maintained in culture medium supplemented with 10% FBS and anti‐CD28 antibody (2 µg/mL).

### 2.12 RNA Interference and Lentiviral Transduction

Small interfering RNA (siRNA) transfection was performed using the riboFECT CP transfection reagent. CD4^+^ T cells were seeded in 24‐well plates containing RPMI‐1640 medium. Transfection complexes were prepared by mixing 1×riboFECT CP buffer, 20 µm PKM2 siRNA, XBP1 siRNA, or scrambled siRNA. Then, RiboFECT CP was added. After incubation, the transfection complex was added to the cells. The cells were then maintained at 37°C and 5% CO_2_. The knockdown efficiencies were validated using quantitative real‐time PCR. For lentiviral transduction, CD4^+^ T cells were mixed with lentivirus in the presence of 8 µg/mL polybrene. The culture plates were sealed and subjected to spinoculation by low‐speed centrifugation (1200 × *g*) for 1 h at 30°C. Afterward, the cells were transferred to a standard incubator for further culture.

### 2.13 RNA Isolation and Quantitative Real‐Time PCR (qRT‐PCR)

Total RNA was extracted using the TRIzol reagent and reverse‐transcribed into cDNA. Afterward, qRT‐PCR was performed following a two‐step PCR reaction procedure. The data were processed using the 2^−(ΔΔcycle threshold) method. The primers are listed in Table S2.

### 2.14 Evaluation of Spleen CD4^+^ T Lymphocyte Polarization and PKM2/H3K9la/XBP1

Phalloidin staining was used to visualize the F‐actin cytoskeleton. The expression levels of pMLC and RhoA were detected using Western blot and immunofluorescence to assess the polarization of CD4^+^ T cells. Briefly, CD4^+^ T cells were seeded on poly‐L‐lysine‐coated coverslips, fixed with 4% paraformaldehyde, and blocked with BSA for 2 h. Cells were then incubated with antibodies against pMLC, RhoA, PKM2, H3K9la, and XBP1s (1:200). The following day, samples were incubated with the corresponding secondary antibodies for 2 h, followed by incubation with phalloidin for 20 min. After rinsing, DAPI anti‐fade mounting medium was applied. Images were observed and captured using a laser scanning confocal microscope (LSM880, Zeiss). ImageJ software was used to quantify the polarization percentages and perform semi‐quantitative fluorescence intensity assessments of pMLC, RhoA, PKM2, H3K9la, and XBP1s.

### 2.15 In Vitro Induction of Primary CD4^+^ T Lymphocytes

After MACS isolation as described above, CD4^+^ T cells were plated at a density of 1.5 × 10^5^ cells per well in 24‐well plates. Recombinant CXCL12 (100 ng/mL) was added, and cells were incubated for 6 h at 37°C.

### 2.16 Cell Migration Assay

A recombinant mouse ICAM‐1/CD54 Fc fusion protein was prepared. Approximately 2 × 10^5^ cells were seeded onto a confocal culture dish pre‐coated with 100 µL of the protein working solution for a 1‐h incubation at 37°C. Finally, images were continuously captured using a laser confocal microscope (LSM880, Zeiss) at 180s intervals for a period of 1 h.

### 2.17 Transwell Assay

Transwell chambers (5.0 µm pore size) were used to evaluate CD4^+^ T cell migration. The upper chambers contained 5.0 × 10^5^ CD4^+^ T lymphocytes in RPMI medium that had undergone the corresponding interventions. The lower chambers were filled with complete medium containing CXCL12 as a chemoattractant. After a 24‐h incubation, migrated cells were fixed and imaged using an inverted microscope (Zeiss), followed by cell counting using ImageJ.

### 2.18 Cleavage under Targets and Tagmentation (CUT&Tag)

CUT&Tag was performed on 100,000 cells using Concanavalin A‐coated beads. Cells were sequentially incubated with anti‐H3K9la antibody overnight at 4°C, secondary antibody for 1 h, and pA‐Tn5 for 1 h at RT. Tagmentation was carried out at 37°C for 1 h with 10 mm MgCl_2_. DNA was purified, and libraries were amplified with 14 cycles of PCR using NEBNext HiFi Master Mix and i5/i7 primers, followed by XP bead cleanup and NovaSeq 6000 sequencing (150 bp PE). Raw reads were aligned using BWA, peaks were called with MACS2 (*q* < 0.05), annotated with bedtools, and subjected to motif (HOMER) and GO/KEGG enrichment analyses. BigWig files were generated with deepTools and visualized in IGV.

### 2.19 RNA‐Sequencing (RNA‐seq)

Total RNA was extracted from BV2 or CD4^+^ T cells using the TRIzol reagent. RNA concentration was measured using Qubit3.0. Following the manufacturer's instructions, the mRNA Library Prep Kit was used to prepare directional RNA sequencing libraries. After library quality assessment, high‐throughput sequencing was performed using a read length of PE150. Differentially expressed genes (DEGs) were determined with a *p*‐value of less than 0.05 and a log2 (fold change) cutoff of 0.585. DEGs were subjected to GO analysis and KEGG enrichment analysis using the Metascape (https://metascape.org/).

### 2.20 Chromatin Immunoprecipitation (ChIP) Assay

Following the manufacturer's protocol, cross‐linking and chromatin digestion were first performed. Then, the chromatin was incubated with anti‐H3K9la, anti‐XBP1s, or control IgG antibodies. Protein A/G magnetic beads were added to the mixture and incubated for 4 h to capture the immunocomplexes. Following the purification of the immunoprecipitated DNA, qRT‐PCR analysis was conducted on the promoter regions of *XBP1*, *TPM2*, *MYO1B*, *KIF4*, kinesin family member protein 17 (*KIF17*), and *PKM2*. The primers used for ChIP‐qPCR are listed in Table S3.

### 2.21 Measurement of the Extracellular Acidification Rate (ECAR)

The Seahorse XFe96 Analyzer (Seahorse Bioscience, Agilent, CA, USA) and Glycolytic Stress Test Kit were used to measure the ECAR. CD4^+^ T cells were plated in assay medium (XF Base Medium containing 2 mm L‐glutamine) and incubated with 10 mm glucose, 1 µm of oligomycin, and 50 mm 2‐deoxy‐D‐glucose (2‐DG). The Seahorse XFe96 software was used to measure and plot the ECAR data. Calculations of the glycolytic parameters were performed in accordance with the Agilent GST user guide [36].

### 2.22 Quantification of Lactate and Pyruvate

Lactate and pyruvate levels were quantified using specific commercial assay kits. Briefly, standard solutions were prepared from stock solutions according to the manufacturer's protocols. Cells were harvested and lysed in the provided extraction buffer. The resulting lysates were centrifuged at 12,000 × *g* for 10 min, and the supernatants were collected for analysis. The Multi‐Mode Microplate Reader was used to record the absorbance (lactate: 570 nm; pyruvate: 520 nm).

### 2.23 Dual‐Luciferase Reporter Gene Assay

To investigate the transcriptional regulation of the *PKM2* promoter by XBP1, wild‐type and mutant PKM2 promoter fragments containing the predicted XBP1 binding motifs were separately cloned into the pGL3‐basic luciferase reporter vector. XBP1 coding sequence was inserted into the pcDNA3.1 vector. HEK293T cells were seeded in 96‐well plates for overnight incubation at 37°C. The following day, the cells were co‐transfected using LipoFiter 3 liposome transfection reagent with the following plasmid combinations: XBP1‐pcDNA3.1+PKM2‐MUT, pcDNA3.1+PKM2‐MUT, XBP1‐pcDNA3.1+PKM2‐WT, pcDNA3.1+PKM2‐WT, along with the internal control vector pRL‐TK. Two days post‐transfection, the luciferase activities were measured. All plasmid constructs were validated via DNA sequencing.

### 2.24 Enzyme‐Linked Immunosorbent Assay (ELISA)

The levels of IL‐1β, IRF8, IL‐6, IL‐33, CXCL11, CXCL10, CXCL9, IL‐36G, TNFRSF8, CCL5, and CCL7 were measured using specific commercial kits following the standard protocols. The concentration of each analyte was determined by interpolation from the corresponding standard curves.

### 2.25 Clinical Samples

All experimental procedures involving human subjects were approved by the First Affiliated Hospital of Zhejiang Chinese Medical University, China (No.: 2022‐KLS‐225‐01). All blood specimens were obtained from the Department of Rheumatology at the First Affiliated Hospital of Zhejiang Chinese Medical University. RA patients with central sensitization were required to meet the 2010 American College of Rheumatology‐European League Against Rheumatism classification criteria, be aged 18 years or older [37], and exhibit a Central Sensitization Inventory (CSI) score ≥ 40. The CSI serves as a screening instrument for symptoms associated with Central Sensitization Syndromes (CSS), which has good structural validity, excellent discrimination ability, and robust test‐retest reliability [38]. CSI scores were stratified into two severity tiers: 40–50 (Low‐CSI) and 51–100 (High‐CSI). Regarding treatment history, all patients had received disease‐modifying antirheumatic drugs (DMARDs) therapy as part of routine clinical management. A total of 30 control subjects, 30 RA patients without central sensitization, and 30 RA patients with central sensitization (15 patients in the Low‐CSI group and 15 patients in the High‐CSI group) were included in this study (Table S4). Peripheral blood specimens were collected using vacutainer tubes. Mononuclear cells were then isolated for subsequent CD4^+^ T cell separation by magnetic bead sorting, following the procedures described above.

### 2.26 Evaluation of PKM2/H3K9la/XBP1 Levels in RA Patients

Peripheral blood CD4^+^ T cells from the enrolled subjects were isolated as described above. The polarization of CD4^+^ T cells, along with the expression of PKM2, H3K9la, and XBP1s, was detected using Western blot and immunofluorescence assays. The correlation between the expression levels of PKM2, H3K9la, and XBP1s and CSI scores was analyzed using Spearman's correlation analysis.

### 2.27 Statistical Analysis

All results were expressed as the mean ± SEM. Statistical analyses between two groups were performed using a 2‐tailed Student's *t*‐test. For multi‐group comparisons, one‐way ANOVA was applied, followed by Dunnett's or Tukey's post hoc tests. The a priori significance value was *p <* 0.05. All data were plotted using the GraphPad Prism 9 program.

## 3 Result

### 3.1 Increased CD4^+^ T Cell Accumulation in the SDH Induces Microglial Activation in CIA Mice

Microglial activation and neuroinflammation within the CNS affect the immune landscape and central pain sensitization in autoimmune disorders, including RA [39]. We therefore performed scRNA‐seq to map the cellular landscape in the spinal cords of CIA mice on day 42 post‐initial immunization, aiming to determine the mechanism underlying chronic pain in experimental arthritis (Figure 1A; Figure S1). Based on cell‐specific gene markers (Figure 1B–D), we annotated the clusters into distinct cell subsets, in which T cells were detected in the spinal cord of CIA mice and established a complex cellular communication network with resident glial cells, particularly microglia (Figure 1E–H).

**FIGURE 1:**
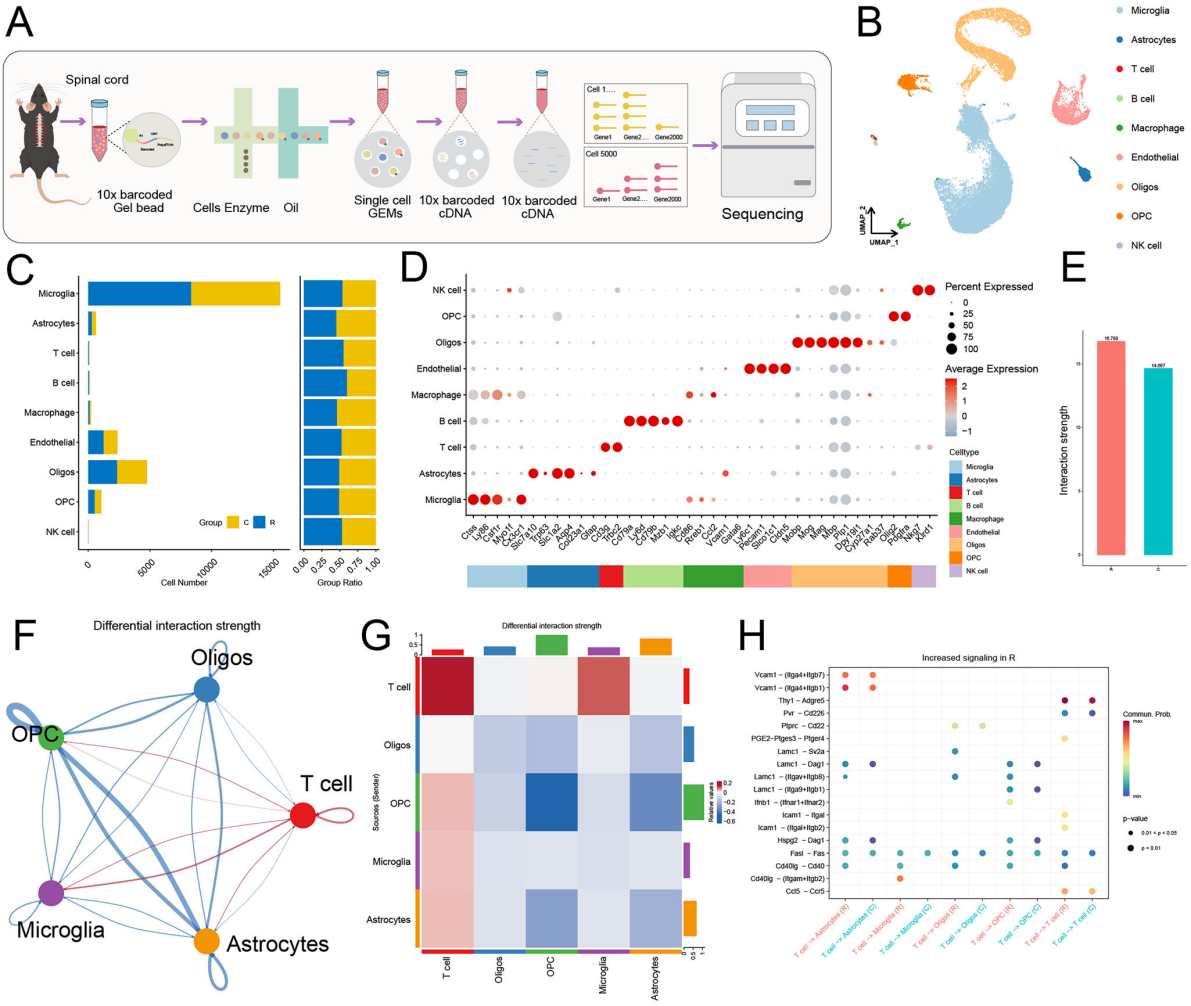
Single‐cell RNA sequencing (scRNA‐seq) reveals a complex cellular communication network between T cells and microglia in the spinal cord of CIA mice. (A) Schematic workflow of the scRNA‐seq analysis of spinal cords from control and CIA mice. (B) UMAP visualization identifying nine distinct CNS cell clusters across all tissue regions and subjects. (C) Sample type fractions relative to the total cell count per cell type. (D) Expression matrix of marker genes across the nine identified cell clusters. (E) Differential interaction strength between the control and CIA groups. (F) Network graph of cellular interaction strength. Red lines indicate an increase in interactions between the CIA and control groups, whereas blue lines indicate a decrease in interactions. The thickness of the lines represents the strength of the interactions. Compared to the control group, interactions between T cells and microglia in the CIA group are markedly enhanced. (G) A strength heatmap of the cellular interactions. The more intense the red color in the heatmap, the stronger the interaction. The interactions between T cells and microglia in the CIA group are more active than in the control group. (H) A bubble chart of the cellular interactions. The chart indicates that in the CIA group, interactions between T cells and other cell types are upregulated compared to the control group. C: Control group, R: CIA group.

Furthermore, we investigated whether CD4^+^ T or CD8^+^ T cells preferentially accumulate in the SDH and explored their potential pathogenic role in activating microglia in CIA mice. Western blot experiments indicated that the CD4 protein levels were significantly increased in the SDH of CIA mice (Figure S2A,B). Furthermore, flow cytometry confirmed a significant increase in the accumulation of CD4^+^ T cells in the SDH of CIA mice (Figure S2C,D). Microglia in these mice exhibited a distinct amoeboid morphology, characterized by a loss of ramification, which was accompanied by increased production of IRF8 and IL‐1β, as indicated by immunofluorescence colocalization (Figure 2A,B). CD4 immunofluorescence intensity was positively correlated with that of IBA1 (Figure 2C,D; *R* = 0.75 and *p* = 0.013). The above findings indicated a potential positive correlation between CD4^+^ T cell accumulation and microglial activation in arthritic chronic pain.

**FIGURE 2:**
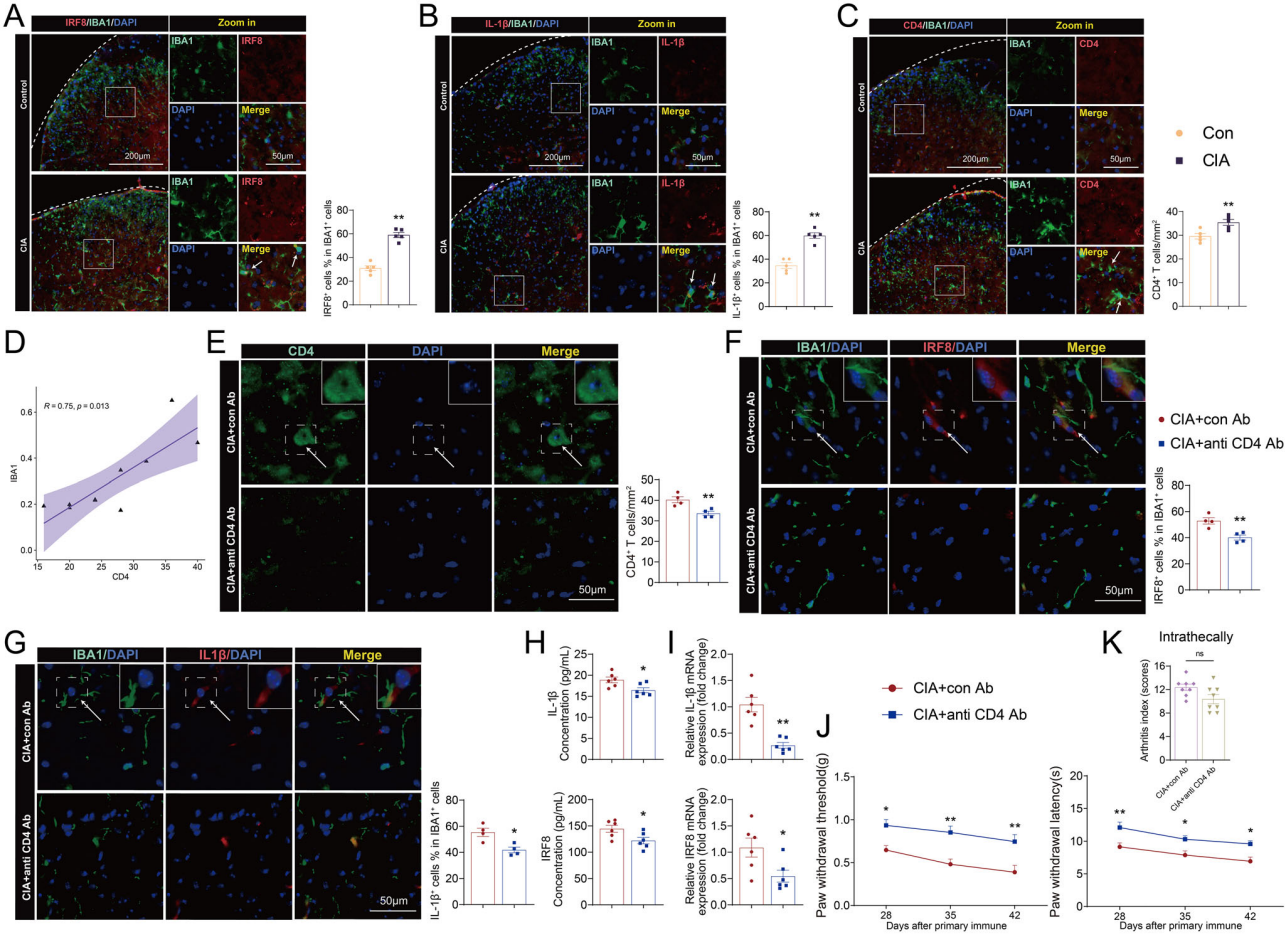
Increased CD4^+^ T cell accumulation in the SDH of CIA mice stimulates microglial activation. (A–C) Double immunofluorescence staining of IRF8 with IBA1 (A), IL‐1β with IBA1 (B), and CD4 with IBA1 (C). Scale bar: 200 µm (left), 50 µm (right); the white arrows indicate highly defined amoeboid morphology microglia (*n* = 5 per group). (D) The correlation between the expression of CD4 and IBA1. (E) The immunofluorescence staining of CD4. Scale bar: 50 µm (*n* = 4 per group). (F, G) The double‐immunofluorescence staining of IRF8 and IBA1 (F), as well as IL‐1β and IBA1 (G). Scale bar: 50 µm (*n* = 4 per group). (H, I) ELISA and qRT‐PCR results revealing the concentration (H) and mRNA expression (I) of IL‐1β and IRF8 following antibody treatment (*n* = 6 per group). (J) Mechanical pain threshold and thermal withdrawal latency in CIA mice treated with or without an anti‐CD4 Ab (*n* = 8 per group). (K) Arthritis index in CIA mice intrathecally treated with or without an anti‐CD4 antibody. (*n* = 8 per group). ns: no significance; ^*^ *p <* 0.05 and ^**^ *p <* 0.01 vs. Control or CIA+con Ab group; Student's two‐tailed *t*‐test or two‐way ANOVA.

To determine the causal role of spinal accumulated CD4^+^ T cells in microglial activation and central sensitization‐like behaviors in vivo, neutralizing antibodies against CD4 were administered. Immunofluorescent staining indicated a significant decrease in CD4^+^ T cell accumulation in the SDH of CIA mice following antibody administration (Figure 2E). Concurrently, a decrease in IRF8 and IL‐1β expression within IBA1‐positive cells was observed (Figure 2F,G). ELISA and qRT‐PCR further indicated significantly decreased concentrations of IL‐1β and IRF8, as well as their mRNA expression in SDH tissues of CIA mice with antibody administration (Figure 2H,I). Behavioral and pathological assessments further demonstrated that the neutralization of CD4^+^ T cells significantly alleviated mechanical and thermal pain hypersensitivity in mice (Figure 2J). To determine whether the effect of CD4^+^ T cell infiltration on central sensitization is independent of arthritis severity, mice were administered intrathecal injections of CD4‐neutralizing antibodies. The results indicated that intrathecal delivery of anti‐CD4 antibodies failed to alter arthritis scores in mice (Figure 2K). Therefore, spinal CD4^+^ T cells may modulate central pain processing independently of peripheral joint inflammation.

For in vitro investigation, we cultured the BV2 microglial cell line with blank medium or conditioned medium from CD4^+^ T cells (CD4^+^ CDCM) to determine if CD4^+^ T cells play a role in mediating microglial activation. Subsequently, BV2 cells subjected to these treatments underwent transcriptomic profiling. RNA‐seq indicated a significant increase in the expression of pro‐inflammatory genes in BV2 cells induced by CD4^+^ CDCM (Figure S3A,B). KEGG enrichment analysis indicated that the DEGs induced by CD4^+^ CDCM were mainly linked to classical immune activation and recruitment pathways, including the NF‐κB Toll‐like receptor and TNF signaling pathways (Figure S3C). Furthermore, the top ten differentially expressed pro‐inflammatory factors (based on |logFC| values) were visualized using bar plots (Figure S3D). In vitro ELISA further confirmed a significant increase in the secretion of these ten pro‐inflammatory factors in CD4^+^ CDCM‐treated BV2 cells (Figure S3E). CD4^+^ CDCM treatment also significantly elevated the fluorescence intensity of INOS in BV2 cells (Figure S3F). Moreover, intrathecal injection of CD4^+^ T cells into control mice resulted in a pronounced upregulation of these ten pro‐inflammatory factors in the SDH tissues (Figure S3G). Overall, these results suggested that aberrant accumulation of CD4^+^ T cells in the SDH may drive microglial activation, thereby contributing to central sensitization‐like behaviors and sustaining chronic arthritic pain.

### 3.2 Lactate Derived from PKM2‐Mediated Glycolysis Contributes to CD4^+^ T Cell Trafficking to the SDH in CIA Mice

Metabolic reprogramming represents a hallmark of activated T cells [40]. Abnormal metabolic programs, including dysregulated glycolysis and oxidative phosphorylation, disrupt the immunoregulatory function of T cells and lead to chronic inflammation [41]. Therefore, to clarify the effect of the metabolic profile on circulating CD4^+^ T cell trafficking to the SDH in CIA mice, we performed LC‐MS/MS metabolomics analysis on splenic CD4^+^ T cells isolated from CIA and control mice. Under both positive and negative ionization modes, samples from the two groups showed a distinct cluster separation (Figure S4A,B). The volcano plot and heat map indicated that various metabolites of CD4^+^ T cells from CIA mice significantly differed from those of control mice (Figure 3A,B). Furthermore, these differential metabolites were associated with glycolysis/gluconeogenesis, HIF‐1, and pyruvate metabolism (Figure 3C). Consistent with this finding, our results also indicated that the level of lactate was significantly increased in CIA mice, based on a global untargeted metabolomics analysis (Figure 3D). Furthermore, enhanced glycolysis was observed in the CD4^+^ T cells of CIA mice, as indicated by an increase in glycolytic capacity (Figure 3E) and an elevation in lactate concentration in spleen CD4^+^ T cells (Figure 3F). To further identify the effects of lactate on CD4^+^ T cells at the transcriptome level, RNA‐seq was conducted on CD4^+^ T cells cultured in the presence or absence of exogenous lactate (Figure 3G,H). Enrichment analysis indicated that following incubation with lactate, the DEGs were mainly enriched in pathways associated with the positive regulation of leukocyte migration, regulation of actin binding, and leukocyte transendothelial migration, indicating that lactate facilitated the migration of CD4^+^ T cells (Figure 3I).

**FIGURE 3:**
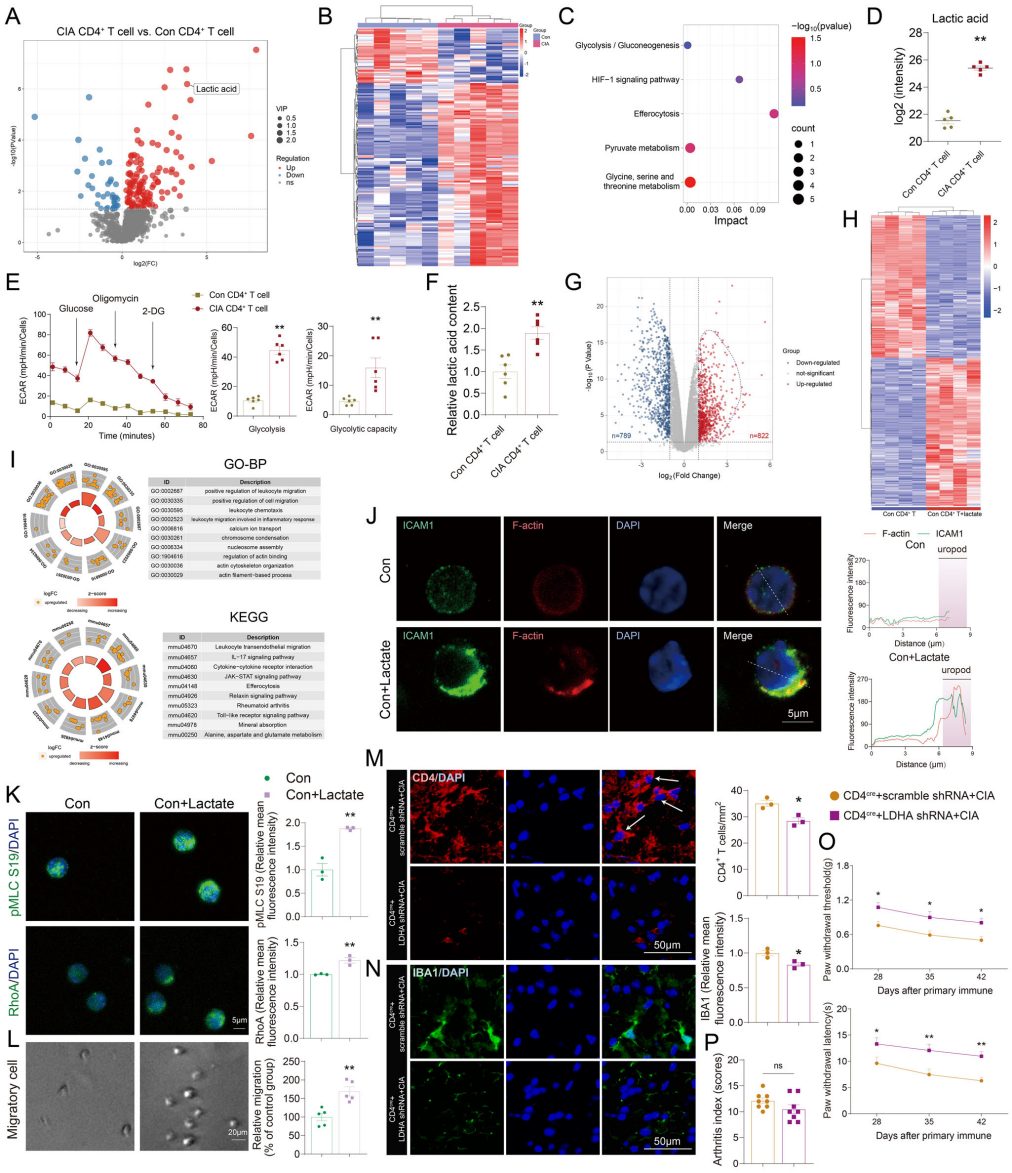
Lactate‐mediated glycolysis contributes to the migration of CD4^+^ T cells into the CNS of CIA mice. (A, B) The volcano plot (A) and heat map (B) of differential metabolites between the control and CIA groups. The metabolites that were increased are marked in red. The metabolites that were decreased are marked in blue. (C) A bubble map of the impact values for the metabolic pathways. (D) The levels of lactate between CD4^+^ T cells derived from control and CIA mice, based on global untargeted metabolomics (*n* = 5 per group). (E) A representative analysis of extracellular acidification rate (ECAR) of CD4^+^ T cells derived from control and CIA mice (*n* = 6 per group). (F) The lactate concentration in CD4^+^ T cells derived from control and CIA mice was detected using a lactate content assay kit (*n* = 6 per group). (G, H) Volcano plot (G) and Heat map (H) of differentially expressed genes in CD4^+^ T cells incubated with or without lactate. (I) GO‐BP and KEGG enrichment analysis of the differentially expressed genes in CD4^+^ T cells incubated with or without lactate. (J) CD4^+^ T cells incubated with or without lactate were stained for the indicated proteins (F‐actin and ICAM‐1) and imaged by confocal microscopy; the uropod is marked by ICAM‐1 (left). Fluorescence intensity quantification analysis was performed from the uropod to the leading edge over the line represented in the merged image (right). Scale bar: 5 µm. (K) Confocal laser fluorescence microscopy was used to determine the level of pMLC S19, and RhoA. Scale bar: 5 µm (*n* = 3 per group). (L) A transwell assay indicating the number of migrated CD4^+^ T cells incubated with or without lactate. Scale bar: 20 µm (*n* = 5 per group). (M, N) Immunofluorescence detection of CD4 (M) and IBA1 (N) in CD4‐Cre mice following shRNA (AAV) treatment and CIA modeling. Scale bar: 50 µm (*n* = 3 per group). (O, P) Mechanical pain threshold and thermal withdrawal latency (O), as well as arthritis index (P) in CD4‐Cre mice following shRNA treatment and CIA modeling (*n* = 8 per group). ns: no significance; ^*^ *p <* 0.05 and ^**^ *p <* 0.01 vs. Con or Control CD4^+^ T cell or CD4^Cre^+scramble shRNA+CIA group; Student's two‐tailed *t*‐test or two‐way ANOVA.

Subsequently, we administered oxamate to CIA mice to inhibit lactate production, which led to a significant decrease in the levels of RhoA and phosphorylated myosin light chain (pMLC) S19 in CD4^+^ T cells (Figure S5A,B). RhoA, a well‐established mediator of pMLC, plays a pivotal role in facilitating cytoskeletal reorganization and cell migration [42]. Furthermore, pharmacological inhibition of lactate production via oxamate potently inhibited the central migration of CD4^+^ T cells, decreased the activation of IBA1 in the SDH (Figure S5C,D), and increased the pain threshold in CIA mice, without affecting the arthritis index (Figure S5E,F). In the presence of lactate, there was a significant increase in the polarization of CD4^+^ T cells, morphologically characterized by actin cytoskeleton reorganization (Figure 3J), which occurred in tandem with an increased level of RhoA and pMLC S19 (Figure 3K). Transwell migration assay indicated that cell migration was significantly enhanced by lactate (Figure 3L). Subsequently, we exogenously introduced an shRNA (AAV) targeting LDHA into CD4‐Cre mice to achieve LDHA knockdown (Figure S6A). LDHA deficiency led to significantly decreased spinal accumulation of CD4^+^ T cells, reduced IBA1 activity in SDH, and elevated mechanical withdrawal threshold and thermal withdrawal latency, without affecting arthritis scores (Figure 3M,P). Overall, these results suggested that lactate is not only a metabolic end‐product of glycolysis in CD4^+^ T cells but also plays a vital role in inducing their spinal recruitment potential in the arthritic model.

To obtain deeper insights into the regulatory mechanism of glycolysis in CD4^+^ T cells, the GSE dataset (GSE56649), containing transcriptomic profiles of CD4^+^ T cells from patients with RA and normal controls, was used to find potential DEGs of glycolytic rate‐limiting enzymes in RA (Figure 4A). Differential expression analysis revealed that the expression of two glycolysis‐related genes, *ENO2* and *PKM*, was significantly increased in CD4^+^ T cells from patients with RA (Figure 4B,C). The subsequent qRT‐PCR results indicated a significant increase in the expression of *PKM2* in CD4^+^ T cells of CIA mice (Figure 4D), which aligned with the elevated fluorescence intensity of PKM2 in CD4^+^ T cells within the spleen and SDH of CIA mice (Figure 4E,F). PKM2, one of the rate‐limiting enzymes in glycolysis, is a physiologically irreversible catalyst that converts phosphoenolpyruvate to pyruvate [43, 44]. PKM2 has been previously implicated in facilitating arthritis by promoting metabolic reprogramming of CD4^+^ T cells and lactate accumulation [45]. Therefore, having established the essential role of lactate‐induced CD4^+^ T cell spinal migration in chronic arthritic pain, we further investigated whether PKM2‐orchestrated glycolysis produces lactate to facilitate CD4^+^ T cell migration. To this end, a PKM2 overexpression lentiviral vector was constructed for in vitro manipulation (Figure S6B), which successfully upregulated PKM2 expression in spleen CD4^+^ T cells (Figure S6C,D). Furthermore, the increased expression of PKM2 significantly increased intracellular pyruvate and lactate levels (Figure 4G), and facilitated the polarization and migration of CD4^+^ T cells, as illustrated by the increased levels of RhoA and pMLC S19, and increased migratory cell counts in transwell assays (Figure 4H–J). The above results indicated that lactate, derived from PKM2‐dependent glycolysis, significantly facilitates the spinal recruitment of CD4^+^ T cells in CIA mice.

**FIGURE 4:**
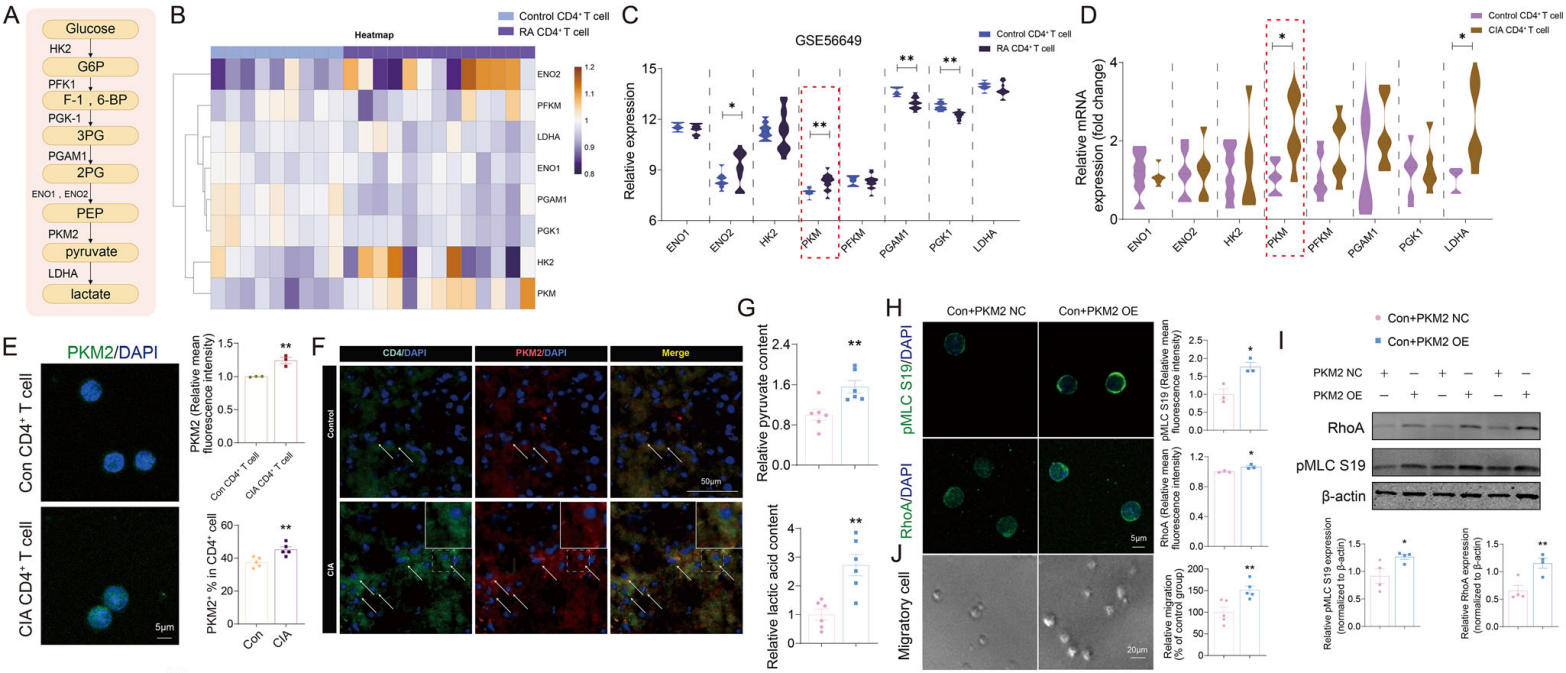
PKM2‐mediated glycolysis contributes to the migration of CD4^+^ T cells into the CNS of CIA mice. (A) Schematic diagram of the key enzymes involved in the glycolysis process. (B, C) A heat map (B) and bar chart (C) of glycolysis‐related enzymes, based on the GSE dataset (GSE56649). (D) qRT‐PCR was used to determine the levels of glycolysis‐related enzymes in CD4^+^ T cells from CIA and control mice; E. Confocal laser fluorescence microscopy was used to detect the level of PKM2 in CD4^+^ T cells. Scale bar: 5 µm (*n* = 3 per group). (F) The double‐immunofluorescence staining of CD4 and PKM2 in SDH. Scale bar: 50 µm (n = 5 per group). (G) The levels of pyruvate and lactate in splenic CD4^+^ T cells (*n* = 6 per group). (H, I) Confocal laser fluorescence microscopy (H; Scale bar: 5 µm; *n* = 3 per group) and western blot (I) *n* = 4 per group) were utilized to determine the levels of pMLC S19 and RhoA in CD4^+^ T cells. (J) A transwell assay showing the number of migrated CD4^+^ T cells. Scale bar: 20 µm (*n* = 5 per group). ^*^ *p <* 0.05 and ^**^ *p <* 0.01 vs. Control CD4^+^ T cell or Control CD4^+^ T cell+PKM2 NC group; by Student's 2‐tailed *t*‐test.

### 3.3 PKM2‐Dependent Lactate Promotes CD4^+^ T Cell Migration Through H3K9la

Since lactate is a precursor for stimulating histone lactylation [27], we further assessed changes in histone lactylation in spleen CD4^+^ T cells from CIA mice. The western blot and immunofluorescence results indicated that the global lactylation levels were significantly increased in both splenic (Figure 5A) and SDH CD4^+^ T cells (Figure 5B) of CIA mice. Interestingly, a 17 kDa protein observed via western blot appeared to be the predominant lactylated protein (Figure 5A). Previous studies have indicated that this prominent ∼17 kDa Pan‐Kla band corresponds to histone H3 [46, 47, 48, 49]. Therefore, we measured site‐specific histone lactylation at H3K9, H3K14, H3K18, and H3K23 (Figure 5C). Similarly, H3K9la levels in splenic and SDH CD4^+^ T cells from the CIA and control groups followed a trend consistent with global lactylation, whereas H3K14la, H3K18la, and H3K23la levels remained unchanged in splenic CD4^+^ T cells (Figure 5C,D). Notably, a significant positive correlation (*R* = 0.78 and *p* = 0.007) was observed between H3K9la levels and CD4^+^ T cells migrating through transwell chambers (Figure 5E). Furthermore, three‐dimensional immunofluorescence reconstruction revealed that H3K9la levels positively correlated with both PKM2 (*R* = 0.97 and *p* < 0.001) and RhoA (*R* = 0.94 and *p* < 0.001) expression, as evidenced by their spatial co‐localization (Figure 5F,G).

**FIGURE 5:**
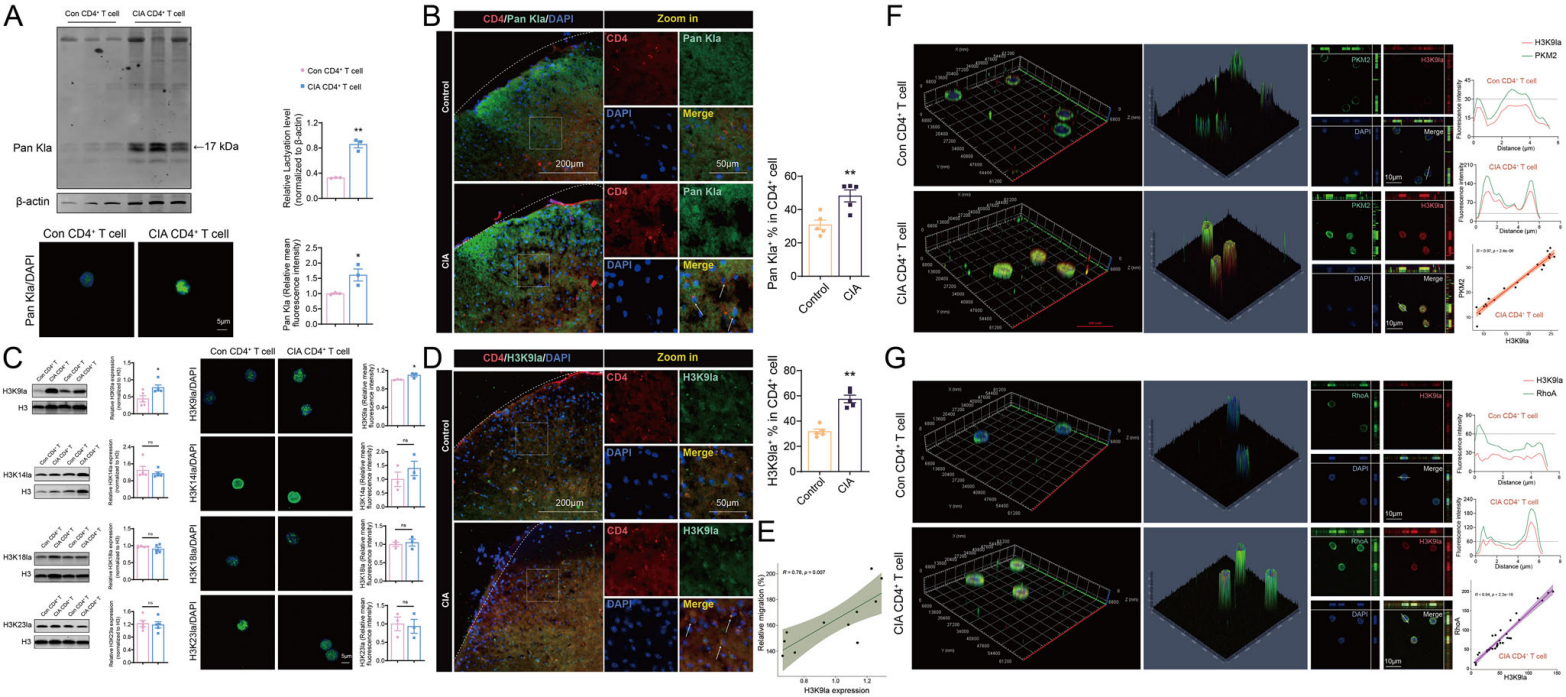
H3K9la is associated with CD4^+^ T cell migration in CIA mice. (A) Global lactylation levels were determined in CD4^+^ T cells in the control and CIA groups by Western blot and immunofluorescence. Scale bar: 5 µm (*n* = 3 per group). (B) The double‐immunofluorescence staining of global lactylation and CD4. Scale bar: 200 µm (left) and 50 µm (right) (*n* = 5 per group). (C) Western blot (*n* = 5 per group) and immunofluorescence (*n* = 3 per group) were used to determine H3K9la, H3K14la, H3K18la, and H3K23la levels in CD4^+^ T cells in the control and CIA groups. Scale bar: 5 µm. (D) Double immunofluorescence staining of H3K9la and CD4. Scale bar: 200 µm (left) and 50 µm (right) (*n* = 5 per group). (E) The correlation between the migration of splenic CD4^+^ T cells and the expression of H3K9la. (F, G) The co‐localization of H3K9la and PKM2 (F), as well as H3K9la and RhoA (G) in CD4^+^ T cells derived from control and CIA groups was analyzed by immunofluorescence, based on a 3D reconstruction technique. Scale bar: 10 µm. ^*^ *p <* 0.05 and ^**^ *p <* 0.01 vs. Con CD4^+^ T cell or Control group, by Student's 2‐tailed *t*‐test.

The overexpression of PKM2 significantly increased H3K9la levels and cell migration in CD4^+^ T cells, both of which were significantly decreased by oxamate (20 mm) [50, 51] treatment in vitro (Figure 6; Figure S7A). In contrast, PKM2 knockdown significantly decreased H3K9la levels and migration in CD4^+^ T cells from CIA mice, an effect that could be rescued by sodium lactate (NaLa) supplementation (Figures S7B and S8). Overall, these results suggested that PKM2‐dependent lactate facilitates CD4^+^ T cell migration by increasing the levels of H3K9la.

**FIGURE 6:**
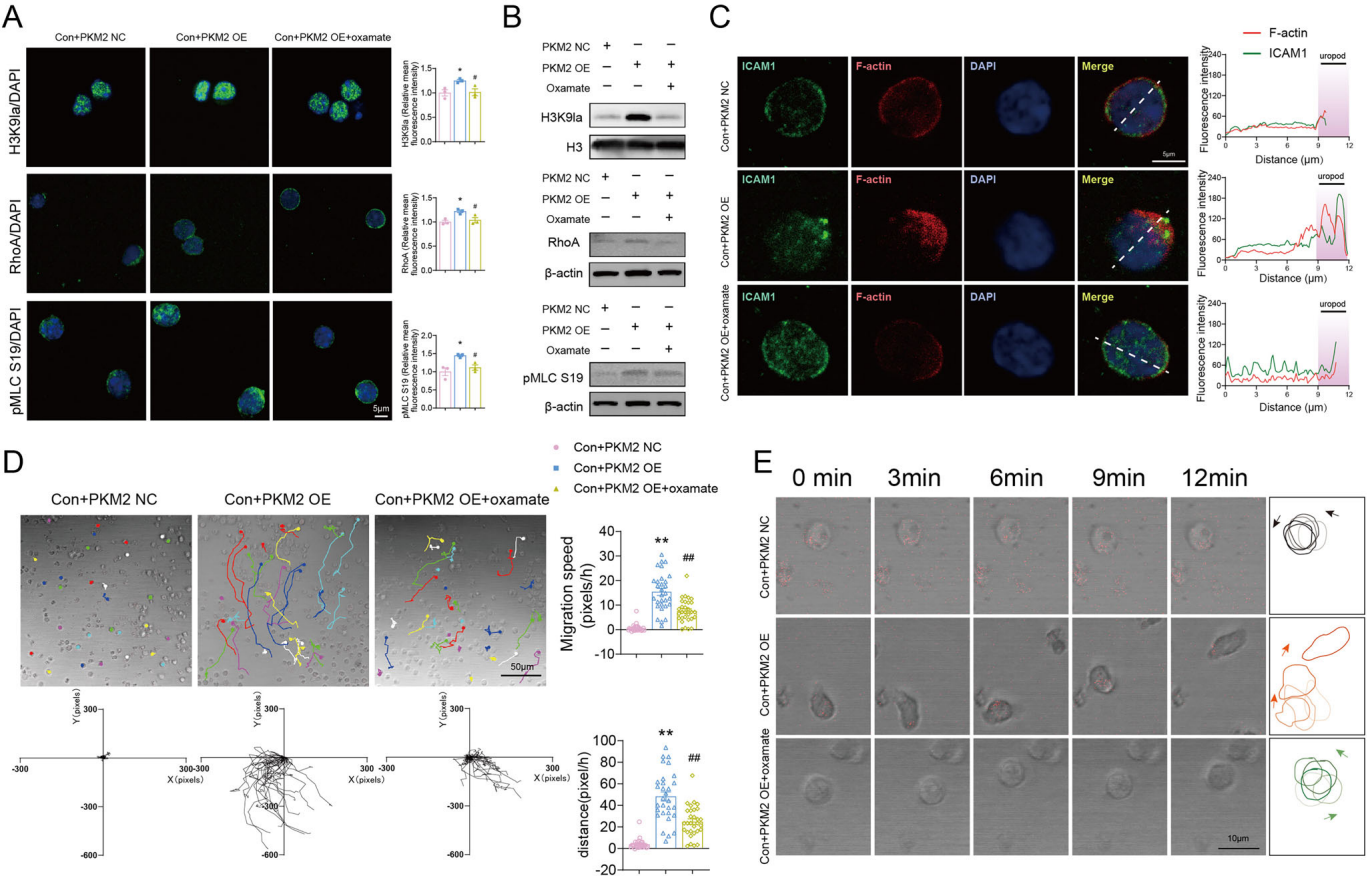
PKM2‐dependent lactate facilitates CD4^+^ T cell spinal recruitment in CIA mice by producing H3K9la. (A) Confocal laser fluorescence microscopy was used to determine the levels of H3K9la, RhoA, and pMLC S19, in the groups. Scale bar: 5 µm (*n* = 3 per group). (B) Western blot was used to determine the levels of H3K9la, RhoA, and pMLC S19. (C) CD4^+^ T cells were stained for F‐actin and ICAM‐1, and imaged by confocal microscopy (left). Fluorescence intensity analysis was performed from the uropod to the leading edge over the line represented in the merged image (right). Scale bar: 5 µm. (D) Quantitative live cell tracking velocity and migration distance of CD4^+^ T cells. Scale bar: 50 µm (*n* = 30 per group). (E) Visual narrative of cell morphology of splenic CD4^+^ T cells, using time‐lapse imaging and tracking, with detailed cell contours presented on the right. Scale bar: 10 µm. ^*^ *p <* 0.05 and ^**^ *p <* 0.01 vs. Control+PKM2 NC group, ^#^ *p <* 0.05 and ^##^ *p <* 0.01 vs. Control+PKM2 OE group, by a one‐way ANOVA, followed by *post hoc* analysis using Dunnett's multiple comparisons test.

### 3.4 PKM2‐Dependent Lactate Increases H3K9la Levels at the Promoter Regions of XBP1 in CD4^+^ T Cells

Histone lactylation can modulate chromatin spatial organization, influence DNA accessibility, and mediate the expression of corresponding genes [52]. To further determine how H3K9la alters gene expression to drive CD4^+^ T cell migration, we performed CUT&Tag analysis using splenic CD4^+^ T cells isolated via magnetic separation (Figure 7A). Using an anti‐H3K9la antibody, a marked enrichment of H3K9la peaks was observed in CD4^+^ T cells of CIA mice compared to the control group (Figure 7B). Comparative analysis further identified 864 up‐regulated and 580 down‐regulated H3K9la binding peaks within promoter regions (Figure 7B,C). Then, the 864 genes with up‐regulated H3K9la binding peaks at their promoters were classified into different KEGG pathways (Figure 7D), among which leukocyte transendothelial migration was one of the most significantly enriched pathways. GO analysis further suggested that the biological functions of these H3K9la target genes were closely related to the molecular functions of regulation of actin cytoskeleton organization, ATPase complex, positive regulation of cell migration, and T cell migration (Figure 7E). These results suggested that H3K9la may play a role in mediating the migration capacity of CD4^+^ T cells in CIA mice.

**FIGURE 7:**
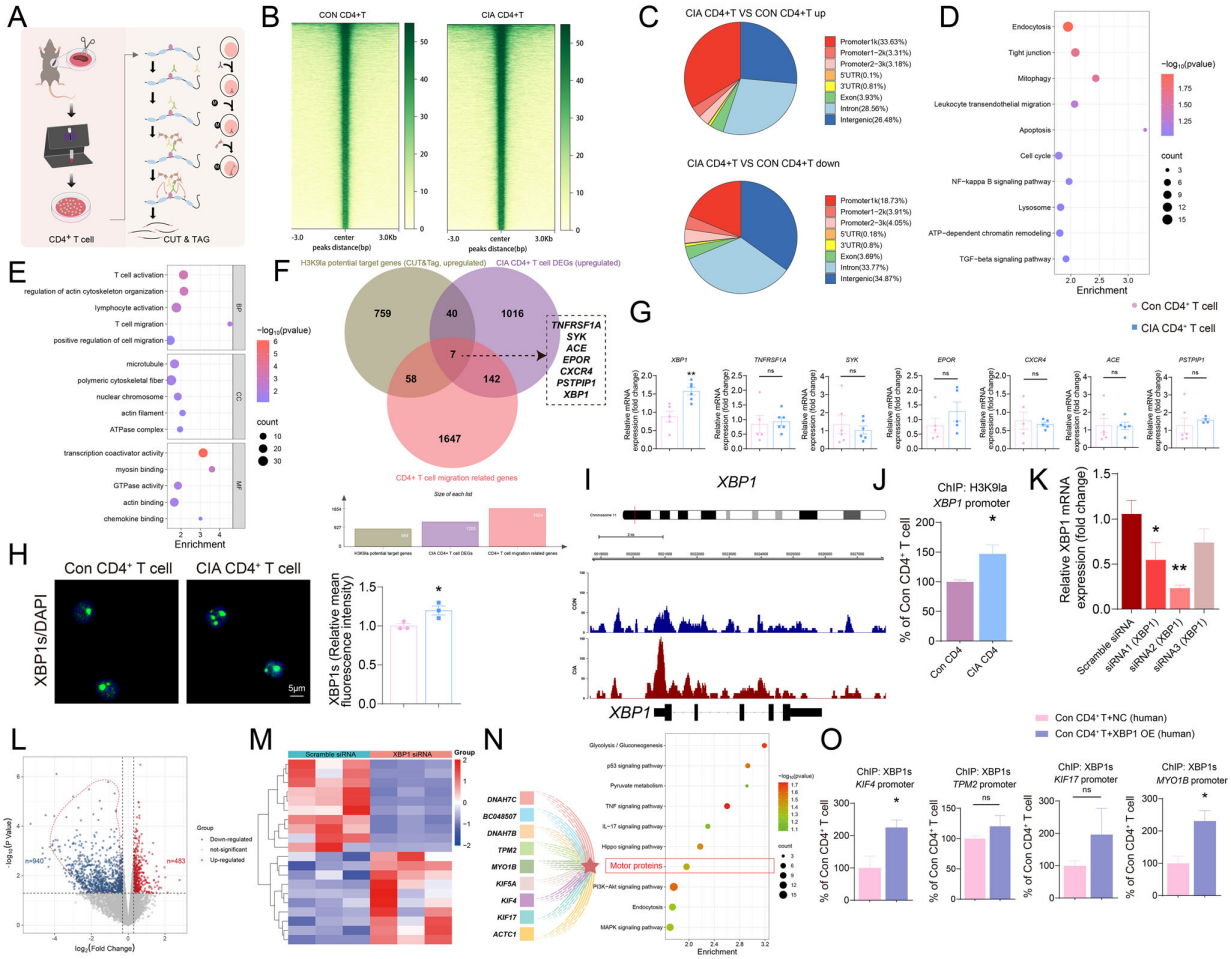
H3K9la is concentrated at the promoters of *XBP1* gene in CD4^+^ T cells of CIA mice. (A) Schematic workflow of the CUT&Tag technique in CD4^+^ T cells, derived from control and CIA mice. (B) The binding density of H3K9la was visualized by deepTools: the heatmap presents the CUT&Tag tag counts for the different H3K9la binding peaks in CD4^+^ T cells, derived from control and CIA mice. (C) Genome‐wide distribution of the up‐regulated and down‐regulated H3K9la‐binding peaks in CD4^+^ T cells, derived from control and CIA mice. (D) KEGG analysis of the H3K9la binding peaks at the gene promoters. (E) GO analysis of H3K9la binding peaks at gene promoters. (F) The intersection of CD4^+^ T cell migration‐related genes with differentially expressed genes (DEGs) in CIA mice containing H3K9la at their promoter regions. (G) qRT‐PCR assays monitoring the mRNA expression of *XBP1*, TNF receptor superfamily 1A (*TNFRSF1A*), spleen tyrosine kinase (*SYK*), erythropoietin receptor (*EPOR*), angiotensin‐converting enzyme (*ACE*), proline‐serine‐threonine phosphatase interacting protein 1 (*PSTPIP1*), and C‐X‐C‐motif chemokine receptor 4 (*CXCR4*) in CD4^+^ T cells derived from control and CIA mice (*n* = 6 per group). (H) Confocal laser fluorescence microscopy was used to determine the level of XBP1s. Scale bar: 5 µm (*n* = 3 per group). (I) Genome browser tracks of CUT&Tag signal at the representative target gene loci. (J) ChIP‐qPCR assay was used to determine H3K9la levels at the *XBP1* promoter regions in CD4^+^ T cells, derived from control and CIA mice (*n* = 3 per group). (K) The effect of XBP1 siRNA was determined by qRT‐PCR. In this study, siRNA2 produced the highest significant effect on the down‐regulation of XBP1, compared with other interference sequences (*n* = 3 per group). (L, M) Volcano plot (L) and heat map (M) of differentially expressed genes between splenic CD4^+^ T cells treated with or without XBP1 siRNA. (N) KEGG enrichment analysis of the differentially expressed genes of splenic CD4^+^ T cells, treated with or without XBP1 siRNA. (O) ChIP‐qPCR assay was used to determine XBP1s levels at the promoter regions of *MYO1B*, *KIF4*, *KIF17*, and *TPM2* in CD4^+^ T cells (*n* = 3 per group). ^*^ *p <* 0.05 and ^**^ *p <* 0.01 vs. Control CD4^+^ T cell, scramble siRNA or NC group, by Student's *t*‐test (two‐tailed) or one‐way ANOVA, followed by post hoc analysis using Dunnett's multiple comparisons test.

To narrow down the potential target genes of H3K9la that regulate CD4^+^ T cell spinal recruitment in CIA mice, we used RNA‐seq to obtain the DEGs in CIA CD4^+^ T cells. We then overlapped the upregulated genes with H3K9la‐modified genes and CD4^+^ T cell migration‐related genes derived from public databases (GeneCards and OMIM). This process yielded seven overlapped genes: *TNFRSF1A*, *SYK*, *EPOR*, *CXCR4, ACE, PSTPIP1*, and *XBP1*, as illustrated by the Venn diagram (Figure 7F). qRT‐PCR indicated that the expression of *XBP1* was significantly increased in the CIA group (Figure 7G), which was consistent with the results of the immunofluorescence assay (Figure 7H). Subsequently, to confirm the direct regulation of XBP1 by H3K9la, we first examined the genome browser tracks of CUT&Tag signals, which visually indicated increased H3K9la enrichment at the *XBP1* promoter region in CIA mice (Figure 7I). To further quantify this observation, ChIP‐qPCR analysis was conducted, revealing that H3K9la enrichment at the *XBP1* promoter was significantly increased in CD4^+^ T cells isolated from CIA mice (Figure 7J).

Next, to determine the specific role of XBP1 in CD4^+^ T cell migration, we performed RNA‐seq to compare the transcriptomes of CIA CD4^+^ T cells with or without XBP1 siRNA transfection (Figure 7K–M). KEGG analysis indicated that the DEGs down‐regulated upon XBP1 silencing were predominantly enriched in pathways mediating cell movement (motor proteins) (Figure 7N). Specifically, nine DEGs were enriched in the motor proteins pathway: *DNAH7C, BC048507, DNAH7B, TPM2, MYO1B, KIF5A, KIF4, KIF17*, and *ACTC1*. According to the JASPAR database, XBP1 is predicted to bind to the promoters of four of these candidate genes: *TPM2, MYO1B, KIF4, and KIF17*. Furthermore, ChIP‐qPCR analysis confirmed that XBP1s binding to the *KIF4* and *MYO1B* promoters was significantly increased in the Control + XBP1 OE group compared to the Control + NC group (Figure 7O). In addition, qRT‐PCR results showed an increase in the mRNA levels of *KIF4* and *MYO1B* in CD4^+^ T cells derived from CIA mice (Figure S9A). *KIF4* and *MYO1B* have been reported to facilitate cell migration via cytoskeletal remodeling [53, 54]. Subsequently, C646, a p300 inhibitor that has been reported to suppress histone lactylation, was applied to CIA mice [55, 56]. The results indicated that C646 significantly attenuated H3K9la, XBP1s, KIF4, and MYO1B expression in splenic CD4^+^ T cells of CIA mice (Figure S9B). Additionally, C646 markedly suppressed the spinal accumulation of CD4^+^ T cells, IBA1 activity, and both mechanical and thermal pain hypersensitivity, without altering the arthritis index (Figure S9C–E). Overall, these results indicated that H3K9la may modulate CD4^+^ T cell migration by facilitating XBP1 signaling.

To determine if H3K9la drives CD4^+^ T cell migration by XBP1 signaling, we silenced XBP1 expression in control CD4^+^ T cells that had been pre‐incubated with NaLa (25 mm) for 24 h. The disruption of XBP1 signaling significantly blunted the lactate‐elicited increase in CD4^+^ T cell migration and mRNA levels of *KIF4* and *MYO1B* (Figure 8A; Figure S10A). Furthermore, immunofluorescence and Western blot experiments confirmed the significant decrease in the protein levels of RhoA and pMLCS19 upon XBP1 knockdown (Figure 8B,C). XBP1 silencing also significantly decreased the polarized morphology induced by lactate incubation (Figure 8D). Live‐cell imaging verified a significant decrease in CD4^+^ T cell migration rate in response to XBP1 silencing (Figure 8E,F). Furthermore, we overexpressed XBP1 in oxamate‐incubated CD4^+^ T cells derived from CIA mice (Figure S10B). The results indicated that the ectopic expression of XBP1 significantly rescued the oxamate‐induced inhibition of CIA CD4^+^ T cell migration, manifested by increases in *KIF4* and *MYO1B* mRNA levels (Figure 8G), RhoA and pMLC S19 protein levels (Figure 8H), and the number of polarized cells (Figure 8I). Subsequently, in vivo rescue experiments were carried out. In CD4^Cre^ mice subjected to CIA, XBP1 overexpression significantly reversed the PKM2 knockdown‐mediated suppression of H3K9la, XBP1s, PKM2, KIF4, and MYO1B levels in splenic CD4^+^ T cells, spinal accumulation of CD4^+^ T cells, and mechanical and thermal pain hypersensitivity (Figure 8J–L). Overall, we concluded that H3K9la facilitates CD4^+^ T cell migration by activating XBP1 signaling.

**FIGURE 8:**
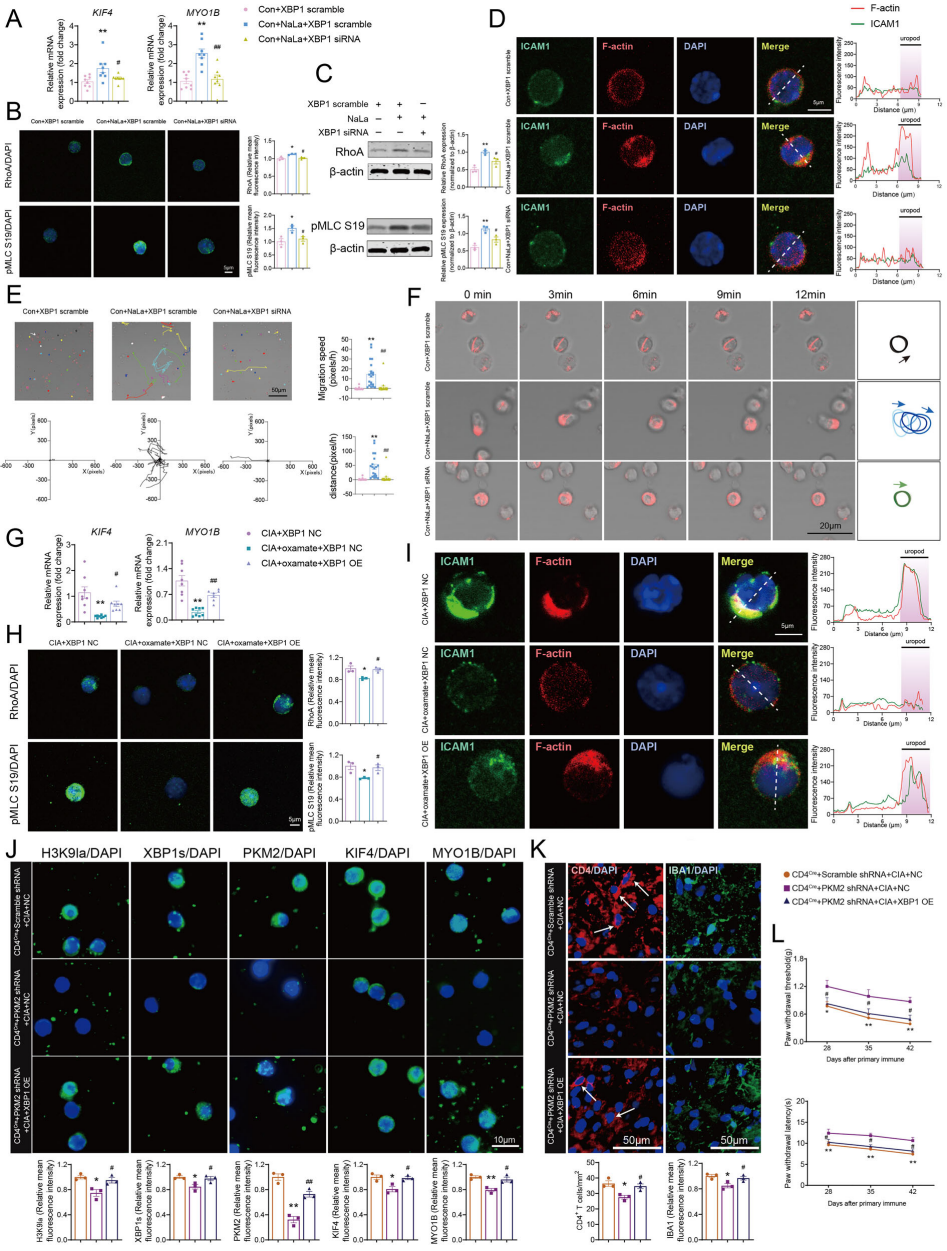
H3K9la facilitates CD4^+^ T cell migration by activating XBP1 signaling. (A) qRT‐PCR assays were used to determine the expression of *KIF4* and *MYO1B* among groups (*n* = 8 per group). (B) Confocal laser fluorescence microscopy was used to determine the level of RhoA and pMLC S19 among groups (*n* = 3 per group). (C) Western blot was used to determine the level of RhoA and pMLC S19 among groups (*n* = 3 per group). (D) CD4^+^ T cells derived from control mice incubated with or without NaLa or XBP1 siRNA were stained for F‐actin and ICAM‐1, and (left) imaged by confocal microscopy; the uropod is marked with ICAM‐1. (right) A fluorescence intensity analysis was performed from the uropod to the leading edge. over the line represented in the merged image. Scale bar: 5 µm. (E) Quantitative live cell tracking of motility indicated a significant decrease in velocities and migration distance of CD4^+^ T cells after incubation with NaLa and XBP1 siRNA, compared with the incubation with NaLa and XBP1 scramble. Scale bar: 50 µm (*n* = 20 per group). (F) A visualization of cell morphology by time‐lapse imaging and tracking, with detailed cell contours presented on the right. Scale bar: 20 µm. (G) qRT‐PCR assays were used to determine the expression of *KIF4* and *MYO1B* in CD4^+^ T cells (*n* = 8 per group). (H) Confocal laser fluorescence microscopy was used to determine the level of RhoA and pMLC S19 among groups. Scale bar: 5 µm (*n* = 3 per group). (I) CD4^+^ T cells derived from spleens of CIA mice incubated with or without oxamate or a XBP1 OE were stained for F‐actin and ICAM‐1, and (left) imaged using confocal microscopy; a uropod is marked with ICAM‐1. (right) A fluorescence intensity analysis was performed from the uropod to the leading edge over the line represented at the merged image. Scale bar: 5 µm. (J) The fluorescence intensity of H3K9la, XBP1s, PKM2, KIF4, and MYO1B among groups. Scale bar: 10 µm (*n* = 3 per group). (K) The immunofluorescence staining of CD4 and IBA1 in SDH tissues among groups. Scale bar: 50 µm (*n* = 3 per group). (L) Mechanical pain threshold and thermal withdrawal latency among groups (*n* = 8 per group). ^*^ *p <* 0.05 and ^**^ *p <* 0.01 vs. Control CD4^+^ T cell+XBP1 scramble or CIA CD4^+^ T cell+XBP1 NC or CD4^Cre^+scramble shRNA+CIA+NC group, ^#^ *p <* 0.05 and ^##^ *p <* 0.01 vs. Control CD4^+^ T cell+NaLa+XBP1 scramble or CIA CD4^+^ T cell+oxamate+XBP1 NC or CD4^Cre^+PKM2 shRNA+CIA+NC group, by a one‐way ANOVA or two‐way ANOVA, followed by post hoc analysis using Dunnett's multiple comparisons test.

### 3.5 PKM2/H3K9la/XBP1 Induces Pain Hypersensitivity by Facilitating CD4^+^ T Cell Trafficking to the SDH in Cia Mice

To further investigate the role of PKM2 in regulating the expression of H3K9la and XBP1 in CD4^+^ T cells, and their subsequent spinal recruitment, we generated CD4‐Cre^+/−^PKM^fl/fl^ mice (CD4^cre^PKM^fl/fl^) for conditional in vivo manipulation of PKM2 in CD4^+^ T cells (Figure 9A; Figure S11A). In CD4^cre^PKM^fl/fl^ mice following CIA induction, the mechanical withdrawal threshold and thermal withdrawal latency were significantly higher compared to the WT group, while the arthritis index remained unchanged (Figure 9B,C). Immunofluorescence and Western blot analyses indicated that PKM2 deficiency significantly decreased the protein levels of H3K9la and XBP1s in splenic CD4^+^ T cells (Figure 9D,E). Concurrently, H3K9la‐ and XBP1s‐positive cells were found to co‐localize with CD4 in the SDH tissue (Figure S11B,C). Interestingly, in response to PKM2 knockout, RhoA and pMLC S19 protein levels in splenic CD4^+^ T cells were also significantly decreased (Figure 9F,G). Similarly, PKM2 knockdown decreased CD4 fluorescence intensity in the SDH of CIA mice (Figure 9H). Furthermore, the number of IBA1‐positive cells and the protein level of IBA1 were significantly decreased, suggesting an inhibition of microglial activation (Figure 9I). Together, our results indicated that the PKM2/H3K9la/XBP1 axis drives the central sensitization‐like behaviors in CIA mice by facilitating CD4^+^ T cell migration into the SDH.

**FIGURE 9:**
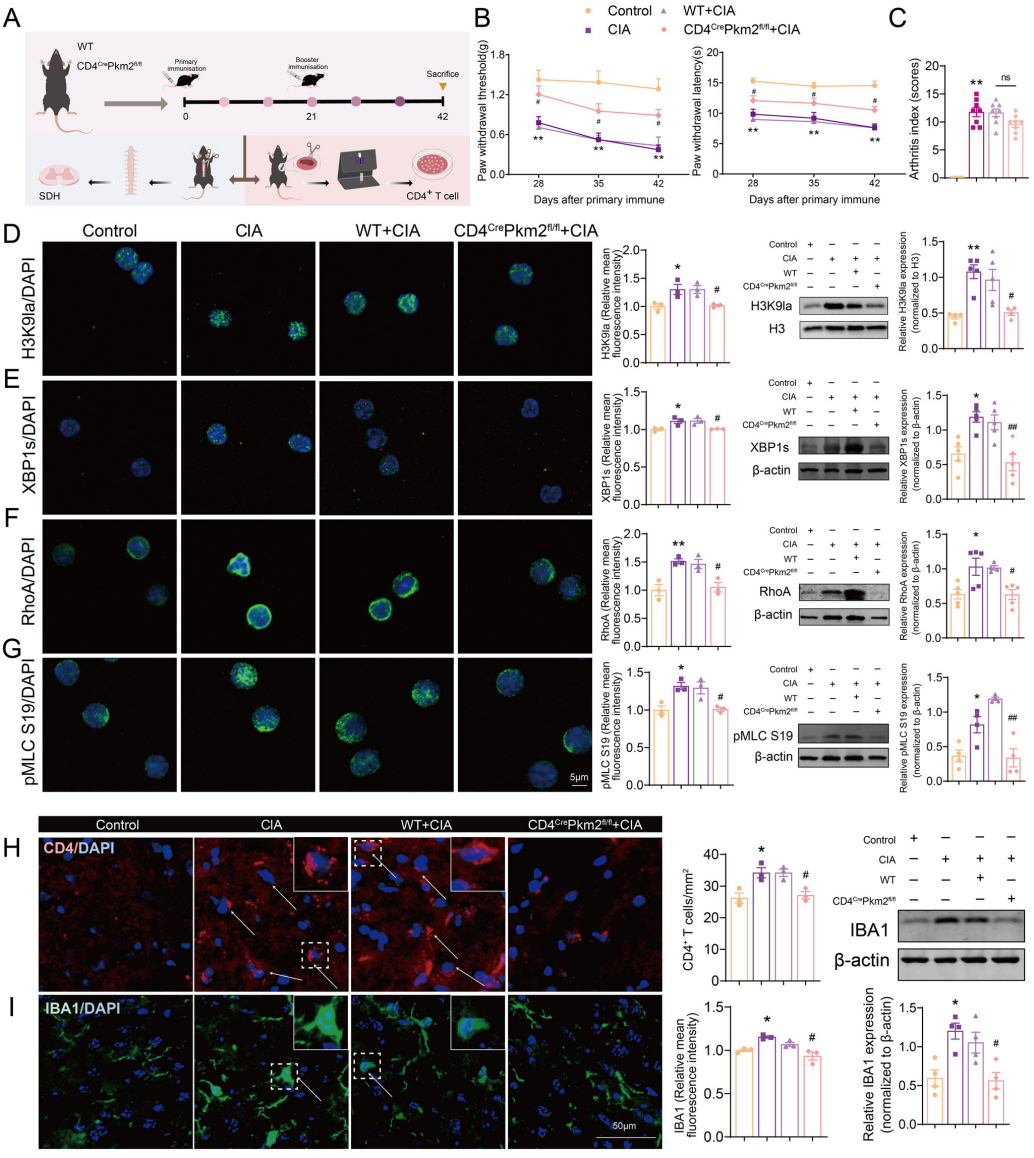
The level of H3K9la/XBP1, central sensitization‐like behaviors, and CD4^+^ T cell spinal accumulation in CD4^cre^PKM^fl/fl^ mice. (A) Schematic illustration of the experiment. (B, C) Mechanical pain thresholds and thermal withdrawal latencies (B), as well as arthritis index (C) in each group (*n* = 8 per group). (D–G) Western blot and immunofluorescence staining were used to determine the levels of H3K9la (D), XBP1s (E), RhoA (F), and pMLC S19 (G) in CD4^+^ T cells in each group. Scale bar: 5 µm (*n* = 3–5 per group). (H) Immunofluorescence staining was used to determine the accumulation of CD4^+^ T cells in the SDH tissue in each group. Scale bar: 50 µm (*n* = 3 per group). (I) Immunofluorescence staining and Western blot were used to determine the levels of IBA1 in the SDH tissue in each group. Scale bar: 50 µm (*n* = 3–4 per group). ^*^ *p <*0.05 and ^**^ *p <* 0.01 vs. Control group, ^#^ *p <* 0.05 and ^##^ *p <* 0.01 vs. WT+CIA group, by one‐way ANOVA or two‐way ANOVA, followed by *post hoc* analysis using Tukey's multiple comparisons test.

### 3.6 PKM2/H3K9la/XBP1 Forms a Positive Feedback Loop That Mediates CD4^+^ T Cell Migration

KEGG analysis predicted that the DEGs in CD4^+^ T cells in response to XBP1 knockdown are mainly involved in the glycolysis and pyruvate metabolic pathways (Figure 7N). We further confirmed the effect of XBP1 on glycolytic processes in CD4^+^ T cells. ECAR analysis revealed that XBP1 silencing significantly decreased cell glycolytic levels and glycolytic function (Figure 10A). To investigate the underlying regulatory mechanism of XBP1 on glycolysis in CD4^+^ T cells, the potential target genes of XBP1 were predicted through bioinformatics, based on JASPAR and the Gene Transcription Regulation Database. The predicted results were intersected with the 940 down‐regulated DEGs mentioned above (Figure 7L), and the resulting Venn diagram highlighted 239 overlapping mRNAs (Figure 10B). Surprisingly, PKM2 emerged as a candidate among these differentially expressed mRNAs in CD4^+^ T cells in response to XBP1 siRNA. Subsequently, bioinformatics analysis, based on the JASPAR database, revealed a potential binding site for XBP1 within the PKM2 promoter region (Figure 10C), indicating that XBP1 may regulate PKM2 transcription. Furthermore, the binding of XBP1s to the PKM2 promoter was verified using the ChIP‐qPCR assay (Figure 10D). Moreover, a dual‐luciferase assay showed that co‐transfection of an XBP1 expression vector with a wild‐type PKM2 promoter construct resulted in significantly higher luciferase activity compared to control vectors. In contrast, co‐transfection with a mutated PKM2 promoter vector abolished this enhancement (Figure 10E). These findings indicated that PKM2 is a direct transcriptional target of XBP1.

**FIGURE 10:**
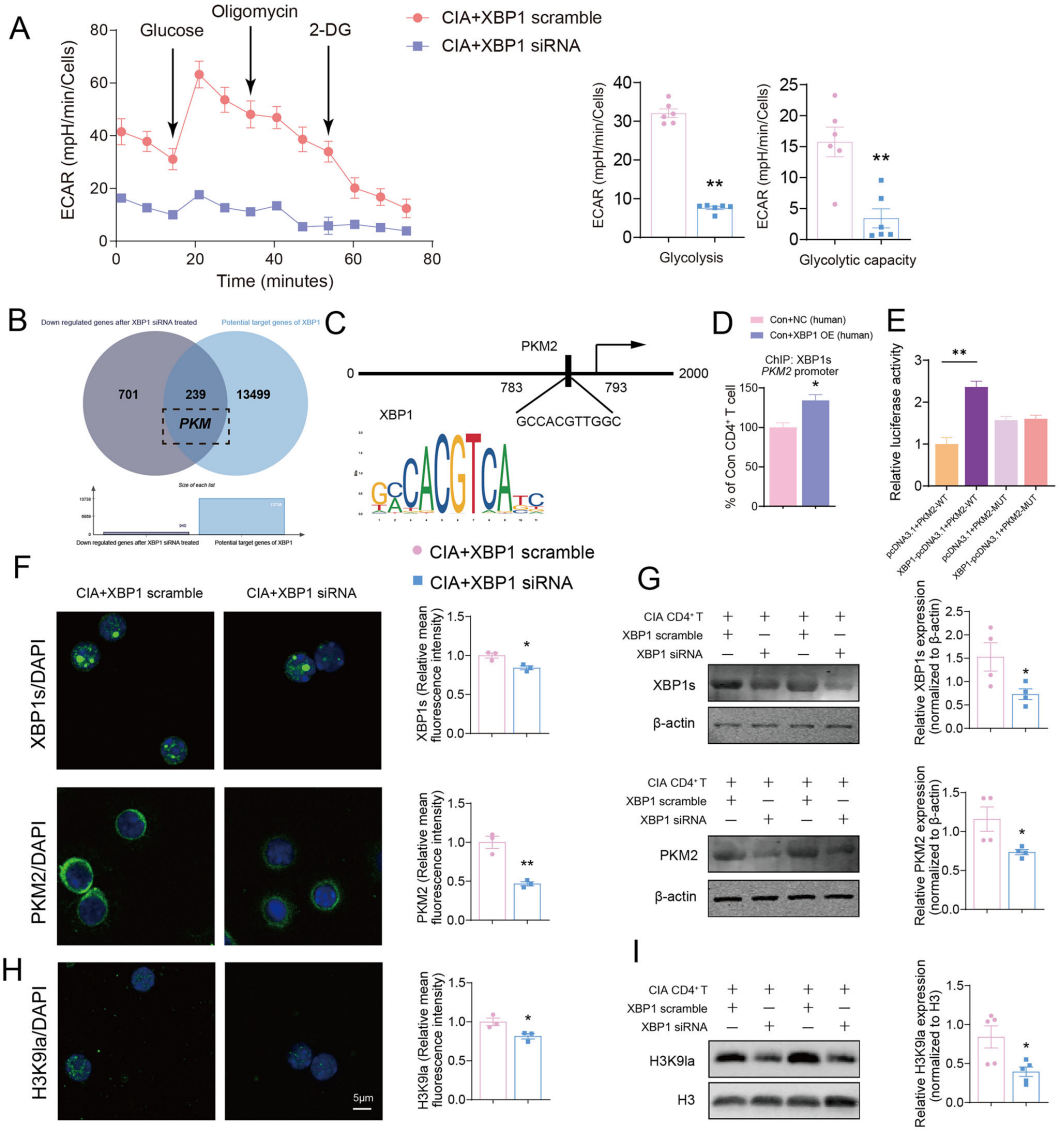
PKM2/H3K9la/XBP1 forms a pathogenic feedback loop that mediates CD4^+^ T cell spinal recruitment. (A) A representative analysis of extracellular acidification rate of splenic CD4^+^ T cells, in the presence of XBP1 siRNA or scramble siRNA (*n* = 6 per group). (B) A Venn diagram showing the intersection of downregulated genes in response to incubation with XBP1 siRNA and the potential target genes of XBP1. (C) The JASPAR database indicated a putative binding site of XBP1 within the promoter region of *PKM2*. (D) ChIP‐qPCR assay was used to determine the levels of XBP1 at the *PKM2* gene promoter regions in CD4^+^ T cells, derived from healthy human (control) peripheral blood (*n* = 3 per group). (E) A dual‐luciferase assay indicated that PKM2 was a direct transcriptional target of XBP1 (*n* = 3 per group). (F) Confocal laser fluorescence microscopy was used to determine the levels of XBP1s and PKM2 between the groups. Scale bar: 5 µm (*n* = 3 per group). (G) Western blot was used to detect the levels of XBP1s and PKM2 between the groups (*n* = 4 per group). (H) Confocal laser fluorescence microscopy was used to determine the levels of H3K9la between the groups. Scale bar: 5 µm (*n* = 3 per group). (I) Western blot was used to determine the level of H3K9la between the groups (*n* = 5 per group). ^*^ *p <* 0.05 and ^**^ *p <* 0.01 vs. CIA CD4^+^ T cell+XBP1 scramble or pcDNA3.1+PKM2‐WT group, by Student's 2‐tailed *t*‐test or a one‐way ANOVA, followed by *post hoc* analysis using Tukey multiple comparisons test.

PKM2 expression was significantly decreased in CIA‐derived CD4^+^ T cells incubated with XBP1 siRNA, as indicated by the immunofluorescence and western blot assay (Figure 10F,G). A similar significant reduction in H3K9la expression was observed in CD4^+^ T cells of CIA mice transfected with XBP1 siRNA (Figure 10H,I). In contrast, XBP1 OE significantly increased the levels of PKM2 and H3K9la in CD4^+^ T cells (Figure S12). Consistent with these findings, our earlier in vivo experiments also revealed that XBP1 overexpression upregulated PKM2 and H3K9la levels in splenic CD4^+^ T cells of CD4^Cre^ mice (Figure 8J). These results indicated that XBP1 signaling stimulates PKM2‐dependent glycolysis and H3K9la, thereby uncovering a positive feedback loop composed of PKM2, H3K9la, and XBP1 signaling.

### 3.7 PKM2/H3K9la/XBP1 Is Involved in the Central Sensitization Syndrome in RA Based on Clinical Samples

CD4^+^ T cells were isolated from the peripheral blood of healthy controls and RA patients with or without central sensitization syndrome. Immunofluorescence indicated that compared to the healthy control group, the polarization of CD4^+^ T cells was substantially increased in RA patients with central sensitization syndrome, as indicated by enhanced fluorescence co‐localization of F‐actin and intercellular adhesion molecule 1 (ICAM1) at the uropod of CD4^+^ T cells (Figure 11A). In addition, immunofluorescence and Western blot analyses indicated that the expression of PKM2, H3K9la, and XBP1s in CD4^+^ T cells from RA patients with central sensitization syndrome was significantly increased compared to those without central sensitization syndrome or healthy controls (Figure 11B–G; Figure S13). Furthermore, the fluorescence intensity of PKM2 (*R* = 0.6 and *p* < 0.001), H3K9la (*R* = 0.59 and *p* < 0.001), and XBP1s (*R* = 0.63 and *p* < 0.001) in peripheral CD4^+^ T cells correlated positively with patient CSI score (Figure 11H–J). We then categorized RA patients with central sensitization syndrome into L‐CSI and H‐CSI groups, using a CSI score of 50 as the cut‐off value. The immunofluorescence intensity of PKM2, H3K9la, and XBP1s in the H‐CSI cohort was significantly higher than that in the L‐CSI cohort (Figure 11K). Together, these results indicated that the PKM2/H3K9la/XBP1 axis may be associated with central sensitization syndrome in patients with RA.

**FIGURE 11:**
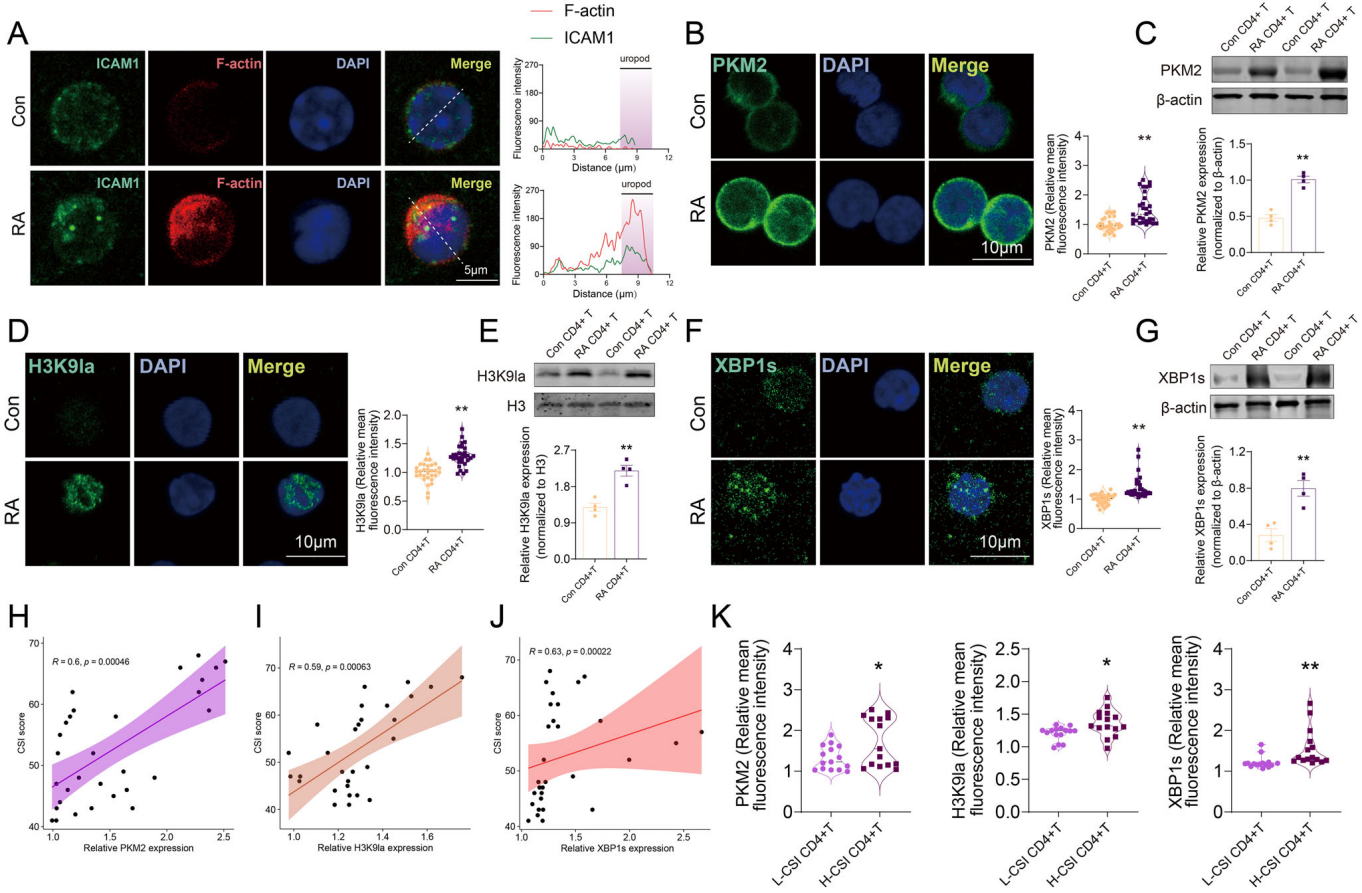
PKM2/H3K9la/XBP1 is involved in mediating the central sensitization syndrome in RA based on clinical samples. (A) CD4^+^ T cells derived from the peripheral blood of control and RA patients with central sensitization syndrome were stained for the indicated F‐actin and ICAM‐1, and (left) imaged by confocal microscopy. (right) Fluorescence intensity analysis was performed from the uropod to the leading edge over the line represented in the merged image. (B, D, F) Confocal laser fluorescence microscopy was used to determine the levels of PKM2 (B), H3K9la (D), and XBP1s (F) in peripheral blood‐derived CD4^+^ T cells from RA patients with central sensitization syndrome and healthy controls (*n* = 30 per group). (C, E, G) Western blot assays were used to determine the levels of PKM2 (C), H3K9la (E), and XBP1s (G) in peripheral blood‐derived CD4^+^ T cells from RA patients with central sensitization syndrome and healthy controls (*n* = 4 per group). (H–J) The correlation between CSI scores and expression levels of PKM2 (H), H3K9la (I), and XBP1s (J) in peripheral blood‐derived CD4^+^ T cells from RA patients with central sensitization syndrome. (K) Confocal laser fluorescence microscopy was used to determine the levels of PKM2, H3K9la, and XBP1s in peripheral blood‐derived CD4^+^ T cells from patients with RA in the L‐CSI and H‐CSI groups (*n* = 15 per group); ^*^ *p <* 0.05 and ^**^ *p <* 0.01 vs. control CD4^+^ T cell group or L‐CSI CD4^+^ T cell group, by Student's 2‐tailed *t*‐test.

## 4 Discussion

Chronic pain represents a primary driver of intractable suffering and disability in RA. To date, it still represents a challenge, as conventional surveillance and management strategies are mainly based on markers of articular and systemic inflammation [57]. Thus, there is an unmet clinical need to elucidate the pain‐producing mechanisms independently of joint inflammation. In this study, we investigated the role of spinal cord accumulation of CD4^+^ T cells in stimulating microglial activation and central sensitization‐like behaviors in the CIA mice. The presented results indicated the central role of a PKM2/H3K9la/XBP1 positive feedback loop in the CD4^+^ T cell trafficking to the SDH of CIA mice. Furthermore, we confirmed an increased level of the PKM2/H3K9la/XBP1 axis in RA individuals and its correlation with central sensitization scores. To our knowledge, this is the first study to comprehensively elucidate the pathological impact and targeted therapeutic potential of glycolysis, histone lactylation, and CD4^+^ T cell spinal recruitment in the chronic pain of RA. Our study may provide a mechanistic foundation for novel strategies aimed at alleviating chronic pain in RA through this pathogenic feedback loop.

In central pain‐processing defects, the importance of microglial activation in chronic pain is well documented [7]. A recent study supports the presence of spinal neuroinflammation in RA [2]. Matsushita et al. [58] reported that microglia are significantly activated in CIA murine models. These findings suggested that microglial activation is a critical determinant of chronic joint pain in RA. Our study, in contrast to prior reports, verified that the increased level of spinal accumulated CD4^+^ T cells, at least in part, underpins microglial activation and microgliosis in this murine model. This finding clearly indicated that the spinal cord is an active site of neuro‐immune interaction in chronic pain pathology, rather than being an immune‐privileged zone [59]. Our results are consistent with a growing body of evidence from neuropathic pain models indicating that T cell‐microglia interactions are fundamental to the establishment of pain hypersensitivity suggestive of central sensitization. For example, specific molecular mediators have been identified, such as the channel protein pannexin‐1, which operates in a sex‐specific manner in microglia and T cells to produce neuropathic pain [60]. Furthermore, the complement component C1q has been reported to mediate microglia‐dependent synapse elimination in pain states, indicating that microglia are a key downstream effector that can be modulated by other cell types and may represent a viable therapeutic target [61]. Similar to our findings, a recent study reported that CD4^+^ αβ T cells selectively infiltrate sites transmitting mechanical allodynia, thereby contributing to the transition from acute to chronic pain following peripheral nerve injury [62]. Proinflammatory CD4^+^ T cells can induce neuroinflammation by infiltrating the CNS and subsequently activating M1 microglia polarization by cytotoxic factors [63]. Cathepsin S‐activated CD4^+^ T cells infiltrate the SDH tissues and secrete IFN‐γ to reactivate microglia, which contribute to the transition of acute pain to a chronic pain state [64]. Tumor cell implantation induces a marked increase in T cell (CD3^+^CD4^+^) numbers and their infiltration into the spinal cord, which promotes microglial activation and exacerbates bone cancer pain [65]. Moreover, an earlier study demonstrated that CD4^+^ T lymphocytes extensively infiltrate the lumbar spinal cord in a rodent spinal nerve L5 transection (L5Tx) model of neuropathic pain, whereas CD4‐deficient mice exhibited significantly attenuated mechanical hypersensitivity following L5Tx [15]. The convergence of these findings across different pain etiologies suggests that the cellular machinery of central sensitization, namely T cell‐microglia crosstalk, may represent a conserved pathophysiological module rather than a feature restricted to specific disease models. This study initially investigated the effects of CNS‐accumulated CD4^+^ T cells on microglial activation in a murine model of RA. Similarly, Yamashita et al. observed that LH2171 administration in mice with CIA suppressed CD4^+^ T cell infiltration into the spinal cord, thereby ameliorating the severity of experimental autoimmune encephalomyelitis [66], but they did not simultaneously track whether microglial activation was affected. Further studies on the molecular cascades for producing CD4^+^ T cell spinal accumulation were of great significance in identifying microglial activation and attenuating pain progression in the context of RA.

Emerging data have implicated metabolic dysregulation in immune cells as a fundamental pathogenic pathway in RA [67]. Studies have confirmed that glycolysis is initiated to meet the increased energy demand and biosynthesis requirements following the activation of tissue‐residing T cells during the intermediate and terminal phase of RA, thus creating a lactate‐enriched microenvironment [68]. Consistent with this, our results indicated that spleen CD4^+^ T cells exhibited enhanced glycolysis driven by the aberrant upregulation of PKM2 in CIA mice, which exerts a profound effect on the spinal recruitment of CD4^+^ T cells. Furthermore, our study confirmed that PKM2‐dependent lactate production stimulates the migration of CD4^+^ T cells in CIA mice. In contrast, a previous study reported that lactate acts as a “stop migration signal” due to its interference with intracellular metabolic pathways [69]. This inconsistency arises from differences in the mechanisms by which lactate affects T cell migration under diverse pathological settings. In addition, our results also reflect, to a certain extent, that the influence of glycolysis on CD4^+^ T cell migration was not only dependent on lactate production. Indeed, lactate deprivation significantly inhibited CD4^+^ T cell migration, whereas the addition of NaLa increased CD4^+^ T cell migration in CIA mice bearing PKM2 knockdown. Lactate has been identified as a signaling molecule and a regulator of gene expression involved in the immune response [70]. This is consistent with our RNA‐seq data indicating that lactate induced broad alterations in the gene expression profile in CD4^+^ T cells, especially genes governing dynamic cytoskeletal remodeling. Thus, these findings supported the hypothesis that lactate derived from PKM2‐driven glycolysis plays a critical role in facilitating CD4^+^ T cell spinal recruitment in CIA mice by modulating the transcriptomic landscape. Similar to our findings, it was reported that in HUT‐78T cells, lactate promotes the production of IL‐4 and IL‐13 through monocarboxylate transporters, thereby modulating T cell activation [71]. Notably, our study represents the first investigation into the impact of glycolysis‐derived lactate on CD4^+^ T cell migration in RA, offering a significant advance to current understanding.

Histone lactylation serves as a metabolic sensor, translating metabolic fluctuations into stable gene expression patterns through a defined modification code [72], thereby positioning lactate at the crossroads between small molecule metabolism and epigenetic regulation. Considering that lactate led to diverse transcriptomic alterations in CD4^+^ T cells associated with cell migration, we hypothesized that histone lactylation is mechanistically involved in CD4^+^ T cell trafficking into the spinal cord in CIA mice. Histone lactylation remains poorly characterized in RA, even though aberrant glycolysis is a significant feature of this disease [73]. In this study, we identified, for the first time, that H3K9la is significantly increased in CD4^+^ T cells from CIA mice. This finding is particularly timely, as histone lactylation is rapidly emerging as a critical epigenetic modification in immunology [74]. A landmark 2024 study reported that H3K9la and H3K18la are dynamically regulated during CD8^+^ T‐cell activation and are critical for their metabolic programming and effector function [74]. While our study focused on CD4^+^ T cells, these parallel findings in CD8^+^ T cells suggested that histone lactylation is a conserved mechanism across T‐cell lineages for translating metabolic states (i.e., high lactate from glycolysis) into functional gene expression programs. Our work extends this paradigm to the context of autoimmunity and neuroinflammation, identifying XBP1 as a key downstream target of H3K9la‐mediated regulation that facilitates a specific cellular behavior: abnormal cell migration.

We used CUT&Tag and RNA‐seq coupled with ChIP‐qPCR validation to establish that XBP1 is a direct downstream target of H3K9la that promotes CD4^+^ T cell migration. XBP1, a basic‐region leucine zipper protein, is ubiquitously expressed across various tissues and participates in the regulation of diverse biological functions [75]. XBP1 has been reported to be significantly overexpressed in RA, where it facilitates the synthesis of pro‐inflammatory cytokines [76, 77]. In addition, XBP1 has been implicated in driving the proliferation of fibroblasts in RA [78]. In this study, we discovered that XBP1 facilitated the accumulation of CD4^+^ T cells within the SDH tissue of CIA mice. Furthermore, we found that XBP1 directly binds to the promoters of *KIF4* and *MYO1B*, thus promoting CD4^+^ T cell migration. *KIF4* is a plus‐end‐directed, microtubule (MT)‐stimulated ATPase [79]. It features a characteristic N‐terminal motor domain that binds to MTs and ATP [80, 81]. *MYO1B* belongs to the molecular motor family and helps to induce cells toward a highly motile phenotype [82]. Previous studies have focused on their effects on cell motility and migration in various types of cancer. For example, KIF4A induces cytoskeletal remodeling and facilitates glioma cell migration [54]. As a molecular motor, *MYO1B* directly influences actin organization and cell morphology, thus facilitating the metastatic phenotype in prostate cancer cells [83]. In this study, we identified the aberrant expression of *KIF4* and *MYO1B* in splenic CD4^+^ T cells of CIA mice, highlighting them as critical downstream effectors of the PKM2/H3K9la/XBP1 signaling axis that facilitates T cell migration. The results of our study have enhanced the understanding of the biological scope of XBP1 signaling in RA by placing it under the control of histone lactylation.

Surprisingly, XBP1 was also found to be enriched in the *PKM2* promoter region, based on data from ChIP‐qPCR and dual luciferase reporter assays, suggesting that XBP1 reciprocally drives PKM2 transcription. This establishes a self‐amplifying positive feedback loop that may explain the persistence of a pro‐migratory, glycolytic phenotype in CD4^+^ T cells. This hypothesis is supported by a recent independent finding in macrophages, where the IRE1α‐XBP1 signaling axis was shown to be necessary for facilitating glycolysis [84]. Our findings in T cells are consistent with these observations in macrophages, suggesting that XBP1‐mediated promotion of glycolysis is a fundamental immunometabolic circuit conserved across diverse immune lineages. In the context of our study, this loop represents a pathogenic cycle: inflammation facilitates PKM2‐dependent glycolysis and lactate production, which induces H3K9la‐mediated XBP1 expression, which in turn augments the glycolytic state by facilitating PKM2 transcription, thereby perpetuating the activation of CD4^+^ T cells and their CNS recruitment potential. This self‐sustaining mechanism may provide a molecular explanation for how central sensitization and chronic pain in RA can become structurally uncoupled from peripheral joint inflammation.

## 5 Conclusion

In summary, our study suggests the presence of a PKM2/H3K9la/XBP1 positive feedback loop that exacerbates CD4^+^ T cell migration to the CNS and stimulates central sensitization‐like behaviors in CIA mice. These findings broaden our understanding of the pathophysiological mechanisms underlying RA‐associated chronic pain and provide a potential therapeutic target for clinical management. However, several limitations should be noted. The migration of CD4^+^ T cells into the SDH remains to be substantiated with more robust and direct evidence. In future studies, it will be essential to employ two‐photon imaging to directly track CD4^+^ T cell migration in real time. Regarding histone modification regulation, it is necessary to further clarify the specific proteins involved in “writing”, “erasing”, and/or “reading” H3K9la, which will increase our understanding of how these modifications affect CD4^+^ T cell migration and chronic pain in RA. In addition, a complex interaction between histone lactylation and other PTMs, such as acetylation, has been proposed [85]. Therefore, further studies on the interaction between PTMs in RA may provide valuable information regarding the pathological mechanisms that mediate chronic pain in RA. Given the exploratory nature of the CSI cutoff used for subgroup stratification, our significant findings between the L‐CSI and H‐CSI groups should be interpreted cautiously and require external validation. Finally, our study did not determine the effects of drugs that can effectively interfere with this feedback loop, due to limitations in clinical sample size and available clinical information. It will be important to evaluate the effect of specific compounds on this pathogenic loop in clinical cohorts with more complete information and larger sample sizes, which may facilitate the development of efficacious treatments for RA and other CD4^+^ T cell‐mediated neuroinflammatory diseases.

## Author Contributions

Y.J.: Conceptualization, Data curation, Formal analysis, Writing – original draft, Writing – review & editing. X.Z.: Data curation, Formal analysis, Investigation, Methodology, Writing – original draft. X.M.: Data curation, Resources, Validation. M.Z.: Formal analysis, Visualization. J.W.: Formal analysis, Validation. Y.L.: Formal analysis, Writing – review & editing. Y.W.: Data curation, Investigation. Y.Z.: Investigation, Methodology. J.Y.: Resources, Visualization. C.R.A.: Review and editing. C.W.: Conceptualization, Project administration, Resources, Writing – original draft, Writing – review & editing. All authors have read and approved the final manuscript.

## Funding

This research was funded by the Zhejiang Provincial Natural Science Foundation of China (Grant No.: LQ24H270006), National Natural Science Foundation of China (Grant No.: 82405298), Zhejiang Traditional Medicine and Technology Program, China (Grant No.: 2024ZF063), Research Project of Zhejiang Chinese Medical University (Grant No.: 2025JKZKTS09) to Y.P.J.; the National Key R&D Program of China (Grant No.: 2022YFC3501204), and Science and Technology Co‐constructed Plan (STD‐ZJ) Project, China (Grant No.: GZY‐ZJ‐KJ‐23009) to C.P.W.

## Ethics Statement

All clinical experimental procedures were approved by the First Affiliated Hospital of Zhejiang Chinese Medical University, China (Approval No.: 2022‐KLS‐225‐01). All animal procedures were performed in accordance with the guidelines approved by the Institutional Animal Care and Use Committee (IACUC) of Zhejiang Chinese Medical University (Approval No.: IACUC‐202311‐26).

## Conflicts of Interest

The authors declare no conflicts of interest.

## Supporting information




**Supporting File 1**: advs78397‐sup‐0001‐SuppMat.docx.

**Supporting File 2**: advs78397‐sup‐0002‐figureS1‐S13.zip.

## Data Availability

The data that support the findings of this study are available from the corresponding author upon reasonable request. The raw multi‐omics data reported in this paper have been deposited in the Genome Sequence Archive in National Genomics Data Center, China National Center for Bioinformation / Beijing Institute of Genomics, Chinese Academy of Sciences (CRA043757, CRA043786, CRA044878, OMIX017232) that are publicly accessible at https://ngdc.cncb.ac.cn/.
