# Supplementary material for: Pyruvate Kinase M2‐Mediated H3K9 Lactylation and XBP1 Signaling Positive Feedback Loop Facilitates CD4+ T Cell Spinal Recruitment and Central Sensitization in Rheumatoid Arthritis
Source: Adv Sci (Weinh). 2026 Nov 9:e78397. Online ahead of print. doi: 10.1002/advs.78397 (PMC13748777; doi:10.1002/advs.78397)
Supplement: Supplementary file 2 [file ADVS-9999-e78397-s002.docx]

**Supplement table 1. Materials used in this study.**

| **REAGENT or RESOURCE** | SOURCE | IDENTIFIER |
| --- | --- | --- |
| **Antibodies** |  |  |
| For CUT&Tag, ChIP: |  |  |
| rabbit anti-L-Lactyl-Histone H3 (Lys9) | PTM BIO | Cat. No: PTM-1419RM |
| rabbit anti-XBP-1s | Cell Signaling Technology | Cat. No: 40435 |
| For immunofluorescence and Western blot: |  |  |
| rabbit anti-XBP1s | Cell Signaling Technology | Cat. No: 40435 |
| mouse anti-XBP1 | Santa | Cat. No: sc-8015 |
| Rabbit anti-L-Lactyl Lysine | PTM BIO | Cat. No: PTM-1401RM |
| rabbit anti-L-Lactyl-Histone H3 (Lys9) | PTM BIO | Cat. No: PTM-1419RM |
| rabbit anti-L-Lactyl-Histone H3 (Lys14) | PTM BIO | Cat. No: PTM-1414RM |
| rabbit anti-L-Lactyl-Histone H3 (Lys18) | PTM BIO | Cat. No: PTM-1406RM |
| rabbit anti-L-Lactyl-Histone H3 (Lys23) | PTM BIO | Cat. No: PTM-1413RM |
| rabbit anti-Histone H3 | Abcam | Cat. No: ab1791 |
| rabbit anti-pMLC (Ser-19) | Cell Signaling Technology | Cat. No: 3671 |
| rabbit anti-RhoA | Abcam | Cat. No: ab187027 |
| rabbit anti-PKM2 | Abcam | Cat. No: ab150377 |
| mouse anti-PKM2 | Santa | Cat. No: sc-365684 |
| rabbit anti-IBA1 | Wako Chemicals | Cat. No: 019-19741 |
| rabbit anti-β-actin | Cell Signaling Technology | Cat. No: 4970 |
| mouse anti-IRF8 | Santa | Cat. No: sc-365042 |
| mouse anti-IL1B | Santa | Cat. No: sc-52012 |
| mouse anti-CD4 | Santa | Cat. No: sc-19641 |
| rabbit anti-CD4 | Cell Signaling Technology | Cat. No: 25229 |
| rabbit anti-CD8 | Signalway Antibody | Cat. No: 53289 |
| goat anti-rabbit IgG (H&L) | LI-COR | Cat. No: 926-32211 |
| goat anti-mouse IgG (H&L) | LI-COR | Cat. No: 926-68070 |
| goat anti-rabbit IgG H&L (Alexa Fluor^®^ 488) | Abcam | Cat. No: ab150077 |
| goat anti-rabbit IgG H&L (Cy3®) | Abcam | Cat. No: ab6939 |
| goat anti-rabbit IgG H&L (Alexa Fluor^®^ 647) | Abcam | Cat. No: ab150083 |
| goat anti-mouse IgG H&L (Alexa Fluor^®^ 488) | Abcam | Cat. No: ab150113 |
| **Chemicals, Oligonucleotides, and Critical commercial assays** | | |
| Immunization Grade Bovine Type II Collagen | Chondrex | Cat. No: 20021 |
| RPMI 1640 | Gibco | Cat. No: 11875119 |
| Fetal bovine serum (FBS) | AusGeneX | Cat. No: FBS500-S |
| recombinant mouse CXCL12/SDF-1α protein | R&D Systems | Cat. No: 460-SD-050 |
| ultra-LEAF™ purified anti-mouse CD3 antibody | Biolegend | Cat. No: 100238 |
| ultra-LEAF™ purified anti-mouse CD28 antibody | Biolegend | Cat. No: 102116 |
| CD4 Monoclonal Antibody (GK1.5) | Thermo Fisher Scientific | Cat. No: 16-0041-86 |
| Protease inhibitor Cocktail | Bimake | Cat. No: B14002 |
| 4% polyformaldehyde | Biosharp | Cat. No: BL539A |
| L-(+)-Lactate | Sigma-Aldrich | Cat. No: L6402 |
| sodium L-lactate | Sigma-Aldrich | Cat. No: 71718 |
| sodium oxamate | Santa | Cat. No: sc-215880A |
| C646 | Selleck Chemicals | Cat. No: S7152 |
| HiFiScript cDNA Synthesis Kit | CWBIO | Cat. No: CW2569M |
| SYBR Green Pro Taq HS Premix | AGBIO | Cat. No: AG11701 |
| Trizol reagent | Invitrogen | Cat. No: 15596026 |
| Lactic Acid (LA) Content Assay Kit | Solarbio | Cat. No: BC2235 |
| Pyruvate Content Assay Kit | Geruisi-Bio | Cat. No: G0870W |
| Seahorse XF Glycolysis Stress Test Kit | Agilent | Cat. No: 103020-100 |
| CoraLite®594-Phalloidin (red) | Proteintech | Cat. No. PF00003 |
| Isoflurane | RWD | Cat. No: R510-22 |
| MACS buffer | Miltenyi | Cat. No: 130-091-221 |
| CD4^+^ T Cell Isolation Kit (mouse) | Miltenyi | Cat. No: 130-104-454 |
| CD4^+^ T Cell Isolation Kit (human) | Miltenyi | Cat. No: 130-096-533 |
| poly-lysine coated slides | LIUSHENG | Cat. No: 188158 |
| Mouse IRF8 ELISA Kit | BYabscience | Cat. No: BY-EM2204982 |
| Mouse IL-1β ELISA Kit | BYabscience | Cat. No: BY-EM220174 |
| Mouse IL-6 ELISA Kit | BYabscience | Cat. No: BY-EM220188 |
| Mouse IL-33 ELISA Kit | BYabscience | Cat. No: BY-EM220184 |
| Mouse CXCL11 ELISA Kit | BYabscience | Cat. No: BY-EM220308 |
| Mouse CXCL10 ELISA Kit | BYabscience | Cat. No: BY-EM220310 |
| Mouse CXCL9 ELISA Kit | BYabscience | Cat. No: BY-EM221809 |
| Mouse IL-36G ELISA Kit | BYabscience | Cat. No: BY-EM223515 |
| Mouse TNFRSF8 ELISA Kit | BYabscience | Cat. No: BY-EM224803 |
| Mouse CCL5 ELISA Kit | BYabscience | Cat. No: BY-EM222421 |
| Mouse CCL7 ELISA Kit | BYabscience | Cat. No: BY-EM221858 |
| riboFECT^TM^ CP transfection reagent | RiboBio | Cat. No: C10511 |
| LV-CMV-3×flag-PGK-Puro-WPRE | BrainVTA | N/A |
| LV-CMV-PKM2-3×flag-PGK-Puro-WPRE | BrainVTA | N/A |
| LV-CMV-XBP1-3×flag-PGK-Puro-WPRE | BrainVTA | N/A |
| PKM2 siRNA, XBP1 siRNA and scramble siRNA | Obio Technology | N/A |
| Chromatin immunoprecipitation (ChIP) assay | Beyotime | Cat. No: P2080 |
| Dual-luciferase reporter gene assay system | Promega | Cat. No: E1910 |
| Transwell plate | Corning | Cat. No: 3421 |
| **Software and microscope** |  |  |
| Image J (version 1.53v) | National Institutes of Health | https://imagej.nih.gov/ij/download.html |
| GraphPad Prism version 9.0 | GraphPad Software | https://www.graphpad.com |
| Confocal laser scanning microscope | Zeiss | LSM880 |
| Fluorescence microscope | OLYMPUS | VS120-S6-W |
| Odyssey fluorescence imaging system | Odyssey | N/A |
| LightCycle 96 real-time PCR | Roche | N/A |

**Supplement table 2. Primers used in the qRT-PCR.**

| **Primers** | **Forward** | **Reverse** |
| --- | --- | --- |
| LDHA-mouse | CGGCTGGGTCCTGGGAGAAC | ACCTCCTTCCACTGCTCCTTGTC |
| PGK1-mouse | AGGGGAAGCGGGTCGTGATG | GGCTCATAAGGACAACGGACTTGG |
| PGAM1-mouse | ACTATGGCGGTCTGACAGGTCTC | ATGGGAGGCGGTGGGACATC |
| PFKM-mouse | ATGAATGCCGCTGTTCGCTCTAC | CCAGACCCTCAAAGCCATCATGTAC |
| PKM-mouse | GTGCCGCCTGGACATTGACTC | TTCAGCCGAGCCACATTCATTCC |
| HK2-mouse | TGCGAATATGGTTGCCTCATCTTGG | TACTGCTCCTCCTCCTCCTCCTC |
| ENO2-mouse | CGGTCACAGAAGCCATCCAAGC | AGTCCGACGACAAGATCAGCAATG |
| ENO1-mouse | CATCCACACCTGACCACCAGAATC | AGTTCCGAGGGCTCCAGACAC |
| MYO1B-mouse | AGGTCTGGCGTGGAGGTTCTG | GCCGCTGCTTCCTTAGGTCTTC |
| KIF4-mouse | GGTGTCCAAGAAGAGCATCCAAGG | GTCCTGGTTCTGATGGCGGTTG |
| CXCR4-mouse | GTCAACCTCTACAGCAGCGTTCTC | CTGACTGTTGGTGGCGTGGAC |
| EPOR-mouse | CTCGCTGTTGCTGACGGTTCTG | TCGCTCTCTGGGCTTGGGATG |
| SYK-mouse | CCAGAGACAGACAAGCAGCAAGAC | CCGATAGCGAAGACCACACAGATG |
| TNFRSF1A-mouse | CCCGCCACTCTCCCTTCCC | CCAGGACCTCGCACCCAGTAG |
| XBP1-mouse | TTGGGCATTCTGGACAAGTTGGAC | ACAGAGAAAGGGAGGCTGGTAAGG |
| ACE-mouse | GGAAGAGCAGAATCAGCGGAATG | GGCTTCATCAGTCTCTAGGTCAATG |
| PSTPIP1-mouse | GGAGGATGGCTGGTGGACTG | GGCGGAGGTATGTGGAGACTG |
| IL-1β-mouse | GTCGCTCAGGGTCACAAGAA | GTGCTGCCTAATGTCCCCTT |
| IRF8-mouse | GAGCTGCAGCAATTCTACGC | CAGTGTCCGGAAACTCCTCC |

**Supplement table 3. The primers used for detecting enriched DNA in the ChIP assay.**

| **Primers** | **Forward** | **Reverse** |
| --- | --- | --- |
| XBP1 | CTTCCACAAGACTCCCAGCTCAAC | TCTTCCGAGAGGCTTCATCCAGTAG |
| TPM2 | AGGATTAGGTGGTTGTGAGACG | GAGGCCTGGATTTTGCAGTG |
| MYO1B | CACACACACCTTCTCACTCAAG | GTTTCCAGATACAGGTCAGTCC |
| KIF4 | GGAGACGCCAACCAATTGC | GACCCTTCCTTCACCTGACC |
| KIF17 | TGGCATGAGAGGAAGCTGTC | TTCTCGAATCCGCACAGTCC |
| PKM2 | ACAAGGTCTGGCTCTGTCAC | AAATTGTCTGGGTGCAGTGG |

**Supplement table 4.** Clinical parameters in healthy control participants and rheumatoid arthritis patients with or without central sensitization^a^

| Parameters | Healthy control (n=30) | Rheumatoid arthritis (without CS) (n=30) | Rheumatoid arthritis (with CS) (n=30) | *P* value |
| --- | --- | --- | --- | --- |
| Age (years)^b^ | 58.50±9.58 | 56.50±12.28 | 57.27±11.42 | >0.05 |
| Sex (male/female)^c^ | 8/22 | 6/24 | 6/24 | >0.05 |
| BMI (kg/m^2^)^b^ | 22.86±2.35 | 23.20±3.09 | 22.05±2.54 | >0.05 |
| Central Sensitization Inventory (score)^d^ | - | 24.10±8.70 | 52.60±8.85 | <0.05 |
| Swollen Joint Count (SJC)^d^ | - | 2.93±1.82 | 3.10±2.02 | >0.05 |
| Physician Global Assessment (MDGA)^d^ | - | 2.95±1.55 | 3.07±1.65 | >0.05 |
| Course of disease^d^ | - | 4.84±1.49 | 5.34±1.58 | >0.05 |

a: Treatment history: All patients had received Disease-modifying antirheumatic drugs (DMARDs) therapy as part of routine clinical management.

b: Data are shown as mean ± SD. Comparisons were made using one-way ANOVA with Tukey’s post hoc test.

c: Data are presented as numbers (n) and analyzed using the chi-square test.

d: Data are expressed as mean ± SD. Comparisons were made using the independent samples t-test.

**Supplement figure 1. Expression of selected marker genes in the major cell types of the spinal cords.**

**Supplement figure 2. Western blot and flow cytometry were used to detect CD4/CD8 expression and CD4^+^ T cell accumulation in the SDH of control and CIA mice.**

A. Western blot was used to detect the expression of CD4 in the SDH of the control and CIA groups (n = 4 per group); B. Western blot was used to detect the expression of CD8 in the SDH of the control and CIA groups (n = 4 per group); C-D. Flow cytometry was used to detect the accumulation of CD4^+^ T cells in the SDH of the control (C) and CIA (D) groups (n = 4 per group). * *P<*0.05 vs. Control group, by Student’s two-tailed *t* test.

**Supplement figure 3. CD4^+^ T cells promote activation and pro-inflammatory factors section in BV2 cells.**

A-B. Volcano plot (A) and heat map (B) of the differentially expressed genes in BV2 cells co-cultured with the blank medium or CD4^+^ CDCM. C. KEGG pathway enrichment analysis of the differentially expressed genes in BV2 cells co-cultured with the blank medium or CD4^+^ CDCM; D. A bar chart of the top ten differentially expressed pro-inflammatory proteins, based on the RNA-seq (n = 3 per group); E. ELISA quantification of ten pro-inflammatory factors in BV2 cells co-cultured with the blank medium or CD4^+^ CDCM (n = 6 per group); F. Fluorescence intensity of INOS in BV2 cells. Scale bar: 10 µm (n = 3 per group); G. ELISA quantification of ten pro-inflammatory factors in SDH tissues of control mice with intrathecal injection of CD4^+^ T cells (n = 6 per group). * *P<*0.05, ** *P<*0.01, and *** *P<*0.001 vs. blank medium or naïve group; Student’s two-tailed t test.

**Supplement figure 4. The total ion chromatogram of metabolites (A) and OPLS-DA score plot (B) of the control and CIA groups.**

**Supplement figure 5. Oxamate inhibits CD4^+^ T cell migration and central sensitization-like behaviors in CIA mice.**

A-B. The confocal laser fluorescence microscopy was utilized to detect the levels of RhoA (A) and pMLC S19 (B) in CD4^+^ T cells. Scale bar: 5 µm (n = 3 per group); C. Immunofluorescence staining was utilized to detect the accumulation of CD4^+^ T cells in SDH tissues. Scale bar: 200 µm (left) and 50 µm (right) (n = 3 per group); D. Immunofluorescence staining was utilized to detect the levels of IBA1 in SDH tissues. Scale bar: 200 µm (left) and 50 µm (right) (n = 3 per group); E-F. Mechanical pain threshold and thermal withdrawal latency (E), as well as arthritis index (F) in CIA mice between groups (n = 8 per group). * *P<*0.05 and ** *P<*0.01 vs. CIA+vehicle group, by Student’s two-tailed *t* test.

**Supplement figure 6. Detection of LDHA and PKM2 expression in CD4^+^ T cells.**

A. Representative immunofluorescence images of LDHA across different groups. Scale bar: 20 µm; B. Representative immunofluorescence images for identifying LV-CMV-PKM2-3×flag-PGK-Puro-WPRE transduced to CD4^+^ T cells. Scale bar: 50 µm; C. Western blot was utilized to detect the expression of PKM2 (n = 5 per group); D. Immunofluorescence was utilized to detect the expression of PKM2. Scale bar: 5 µm (n = 3 per group). * *P<*0.05 vs. Control CD4^+^ T cell+PKM2 NC group, by Student’s two-tailed *t* test.

**Supplement figure 7. The expression of H3K9la in CD4^+^ T cells treated with oxamate or NaLa.**

A. Western blot was utilized to detect the expression of H3K9la in CIA CD4^+^ T cell treated with or without oxamate (n = 4 per group); B. Western blot was utilized to detect the expression of H3K9la in CD4^+^ T cell, following *in vitro* treatment with or without NaLa (n = 5 per group). ** *P<*0.01 vs. CIA or control CD4^+^ T cell group, by Student’s two-tailed *t* test.

**Supplement figure 8. The levels of PKM2, H3K9la, RhoA, and pMLC S19 in siRNA-transfected CD4^+^ T cells with or without NaLa treatment.**

A. The effect of PKM2 siRNA was detected by qRT-PCR. In this study, siRNA3 exhibited the most significant effect on the down-regulation of PKM2 compared with other interference sequences (n = 3 per group); B. The confocal laser fluorescence microscopy was utilized to detect the level of PKM2 between groups. Scale bar: 5 µm (n = 3 per group); C. The confocal laser fluorescence microscopy was utilized to detect the levels of H3K9la, RhoA, and pMLC S19 among groups. Scale bar: 5 µm (n = 3 per group); D. CD4^+^ T cells treated with or without PKM2 siRNA or NaLa were stained for the indicated proteins (F-actin and ICAM-1) and imaged by con-focal microscopy (left). Fluorescence intensity quantification analysis was performed from the uropod to the leading edge over the line represented at the merge image (right). Scale bar: 5 µm. * *P<*0.05 and ** *P<*0.01 vs. CIA CD4^+^ T cell+PKM2 scramble group or scramble siRNA group, ^#^ *P<*0.05 vs. CIA CD4^+^ T cell+PKM2 siRNA group, by Student’s two-tailed *t* test, or one-way ANOVA followed by post hoc Dunnett’s multiple comparisons test.

**Supplement figure 9. The effect of histone lactylation blockade on the H3K9la/XBP1/KIF4/MYO1B axis and spinal recruitment of CD4^+^ T cells in CIA mice.**

A. The mRNA levels of *KIF4* and *MYO1B* in CD4^+^ T cells of control and CIA mice (n = 6 per group); B. The fluorescence intensity of H3K9la, XBP1s, KIF4, and MYO1B in CD4^+^ T cells of CIA mice following C646 treatment. Scale bar: 10 µm (n = 3 per group); C. The fluorescence intensity of CD4 and IBA1 in SDH of CIA mice following C646 treatment. Scale bar: 50 µm (n = 3 per group); D-E. Mechanical pain threshold and thermal withdrawal latency (D), as well as arthritis index (E) of CIA mice following C646 treatment (n = 8 per group). ns: no significance; * *P<*0.05 and ** *P<*0.01 vs. Control, CIA+vehicle group, by Student’s two-tailed *t* test or two-way ANOVA.

**Supplement figure 10. The levels of XBP1s in CD4^+^ T treated with NaLa, oxamate, XBP1 siRNA or XBP1 OE.**

A. The confocal laser fluorescence microscopy was utilized to detect the level of XBP1s among groups (n = 3 per group). Scale bar: 5 µm.; B. The confocal laser fluorescence microscopy was utilized to detect the level of XBP1s among groups (n = 3 per group). Scale bar: 5 µm.; * *P<*0.05 and ** *P<*0.01 vs. Con+XBP1 scramble or CIA+XBP1 NC group, ^#^ *P<*0.05 vs. Con+NaLa+XBP1 scramble or CIA+oxamate+XBP1 NC group, by one-way ANOVA followed by post hoc Dunnett’s multiple comparisons test.

**Supplement figure 11. Western blot detected PKM2 in CD4^+^ T cells (WT vs. CD4^Cre^PKM2^fl/fl^), and double-immunofluorescence assessed H3K9la, XBP1s among groups.**

A. The expression of PKM2 in CD4^+^ T cells between WT and CD4^Cre^PKM2^fl/fl^ groups (n = 4 per group). ** *P<*0.01 vs. WT group, by Student’s two-tailed *t* test. B-C. The double-immunofluorescence staining of H3K9la (B) and XBP1s (C) with CD4 among groups; Scale bar: 50 µm (n = 3 per group). * *P<*0.05 vs. Control group, ^#^ *P<*0.05 vs. WT+CIA group, by one-way ANOVA followed by post hoc Tukey multiple comparisons test.

**Supplement figure 12. The levels of PKM2 and H3K9la in splenic CD4^+^ T treated with or without XBP1 OE.**

* *P<*0.05 vs. Con+XBP1 NC group, by Student’s two-tailed *t* test (n = 3 per group), Scale bar: 5 µm.

**Supplement figure 13. The levels of PKM2, H3K9la, and XBP1 in CD4^+^ T cells from RA patients with or without central sensitization syndrome.**

* *P<*0.05 vs. RA (-CS), by Student’s two-tailed *t* test (n = 30 per group), Scale bar: 10 µm.
